# Supplementary material for: Clickable C-Glycosyl Scaffold for the Development of a Dual Fluorescent and [18F]fluorinated Cyanine-Containing Probe and Preliminary In Vitro/Vivo Evaluation by Fluorescence Imaging
Source: Pharmaceuticals (Basel). 2022 Nov 29;15(12):1490. doi: 10.3390/ph15121490 (PMC9782470; doi:10.3390/ph15121490)
Supplement: Supplementary file 1 [file pharmaceuticals-15-01490-s001.zip › pharmaceuticals-2025954-supplementary.pdf]

Supplementary informations

Clickable *C*-glycosyl scaffold for the development of a dual fluorescent and [^{18}F]fluorinated cyanine-containing probe and preliminary in vitro/vivo evaluation by fluorescence imaging

Julen Ariztia, Kamal Jouad, Valérie Jouan-Hureaux, Julien Pierson, Charlotte Collet, Bertrand Kuhnast, Katalin Selmeczi, Cédric Boura, Sandrine Lamandé-Langle, Nadia Pellegrini Moïse

Preparation of compounds **4**, **5**, **21**

Copy of ^{1}H NMR spectra for compounds **6-20**, **22, 25-27**

Copy of ^{13}C NMR spectra for compounds **6-20**, **26-27**

Copy of ^{19}F NMR spectra for compounds **6**, **8**, **10**, **12**, **14**, **17**, **26**, **27**

Copy of HRMS for compounds **21-22** and **23-24**

Analytical HPLC profile of compound **19** (Figures S1 and S2)

References

3,6-anhydro-2-deoxy-4,5-*O*-(1-methylethylidene)-7-*O*-acetyl-D-*ribo*-heptanoic acid methyl ester 4[1]

To a solution of **3-*E*** and **3-*Z*** (1 g, 3.50 mmol) in ethyl acetate (100 mL), 400 mg of Pd/C 10% w (40% w/w) were added under inert atmosphere. The reaction mixture was stirred at 40 psi of hydrogen atmosphere for 48 h (Paar apparatus). After 48 h, the catalyst was filtered off through a Celite® pad. 400 mg Pd/C 10% w (40% w/w) were added to the filtrate and the mixture was stirred for another 48 h at 40 psi of hydrogen atmosphere. The catalyst was filtered off through a Celite® pad and the filtrate was concentrated under *vacuum*. The crude product was purified by flash chromatography on silica gel (eluent: cyclohexane/EtOAc 100/0 to 50/50) to afford the compound to afford the compound **4**. Quantitative yield, colorless oil, R_f = 0.48 (Cycl/EtOAc: 6/4); **^{1}H NMR ($CDCl_3$, 400 MHz)**: δ (ppm) = 1.34 (s, 3H, CH_3), 1.49 (s, 3H, CH_3), 2.09 (s, 3H, CH_3), 2.72 (dd, 1H, $J_{2a,2b}$= 16.0, $J_{2,3}$ = 5.0 Hz, *H*-2a), 2.81 (dd, 1H, $J_{2b,3}$ = 6.0 Hz, *H*-2b), 3.71 (s, 3H, OCH_3), 4.05 (dd, 1H, $J_{7a,7b}$ = 11.0, $J_{7a,6}$ = 4.5 Hz, *H*-7a), 4.13-4.28 (m, 2H, *H*-7b and *H*-6), 4.36 (m, 1H, *H*-3), 4.67 (d, 1H, $J_{4,5}$ = 6.0 Hz, *H*-5), 4.80 (dd, 1H, *H*-4).

3,6-anhydro-2-deoxy-4,5-*O*-(1-methylethylidene)-7-hydroxy-D-*ribo*-heptanoic acid methyl ester 5[1]

To a solution of **4** (500 mg, 1.70 mmol) in dry methanol (20 mL), a catalytic amount of sodium was added under inert atmosphere. The reaction mixture was stirred for 3 h a room temperature. The mixture was neutralized by addition of Amberlite IR-120 (and filtered. The solvent was removed under *vacuum*, the crude product was purified by flash chromatography on silica gel (eluent: cyclohexane/EtOAc 100/0 to 40/60) to afford the compound **5**. Yield: 85% as a yellow oil, R_f = 0.10 (Cycl/EtOAc: 6/4); **^{1}H NMR (CD_3OD, 400 MHz)**: δ (ppm) = 1.32 (s, 3H, *CH3*), 1.44 (s, 3H, *CH3*), 2.66 (dd, 1H, $J_{2a,2b}$ = 16.5 Hz, $J_{2a,3}$= 7.0 Hz, *H-2a*), 2.72 (dd, 1H, $J_{2b,2a}$ = 16.5 Hz, $J_{2b,3}$= 7.0 Hz, *H-2b*), 3.53 (dd, 1H, $J_{7a,7b}$ = 12.0 Hz, $J_{7a,6}$= 5.5 Hz, *H-7a*), 3.58 (dd, 1H, $J_{7b,7a}$ = 12.0 Hz, $J_{7b,6}$ = 5.5 Hz, *H-7b*), 3.68 (s, 3H, *OCH*3), 4.00 (app td, 1H, $J_{6,7b}$ = $J_{6,7a}$= 5.5 Hz, $J_{6,5}$ = 1.0 Hz, *H-6*), 4.40 (app td, 1H, $J_{3,2a}$ = $J_{3,2b}$ = 7.0 Hz, $J_{3,4}$ = 4.0 Hz, H-3), 4.74 (dd, 1H, $J_{5,4}$ = 6.0 Hz, $J_{5,6}$ = 1.0 Hz, *H-5*), 4.77 (dd, 1H, $J_{4,5}$ = 6.0 Hz, $J_{4,3}$= 4.0 Hz, *H-4*).

Compound 21[2]

To a solution of c(RGDfK) (10.0 mg, 14.0 µmol, 1.0 eq.) in DMF (1mL), 7.1 mg of 6-azidohexanoic acid (28.0 µmol, 2.0 eq.) and 5 µL of Et_3N (35.0 µmol, 2.5 eq.) were added and the mixture was stirred at 30 ºC for 16 h. The organic solvent was evaporated under *vacuum* and the solid residue was washed with diethyl ether. The obtained solid was dried under *vacuum*

to afford the compound **21**. Yield: 75% as white powder. **HRMS** $[M]^+$ m/z = 743.3979 (calculated for $C_{33}H_{51}N_{12}O_8$: 743.3947).

Compound 6 - ^{1}H NMR ($CDCl_3$, 400 MHz)

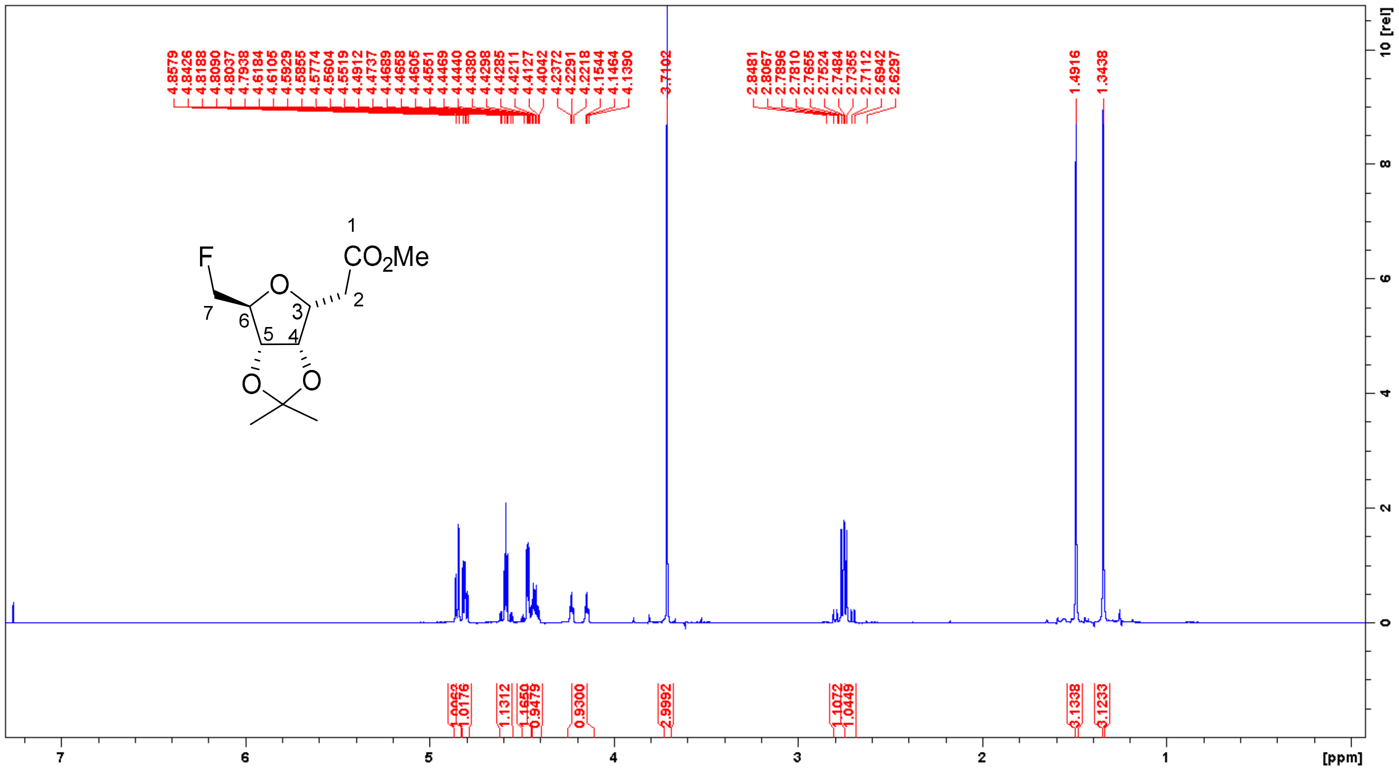

Compound 6 - ^{13}C NMR ($CDCl_3$, 100.6 MHz)

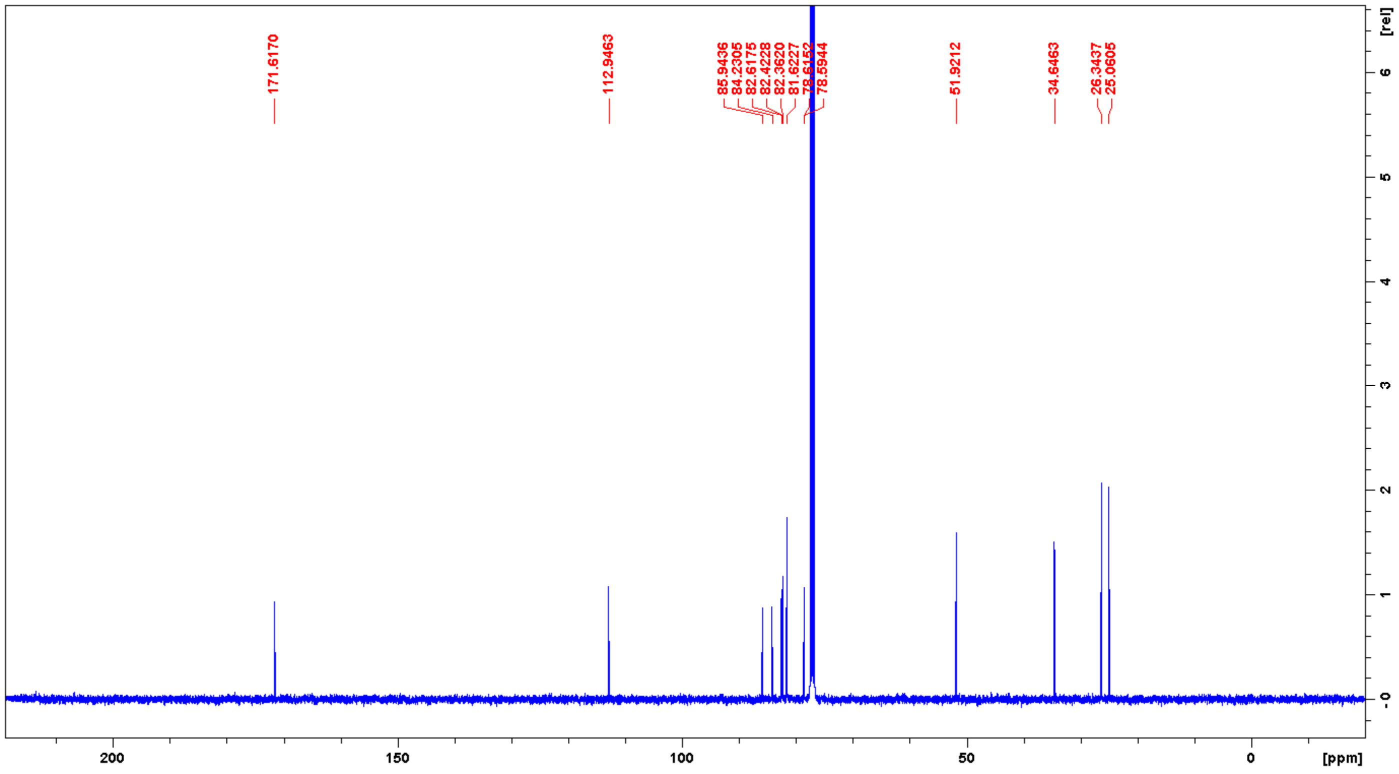

Compound 6 – ^{19}F NMR ($CDCl_3$, 376 MHz)

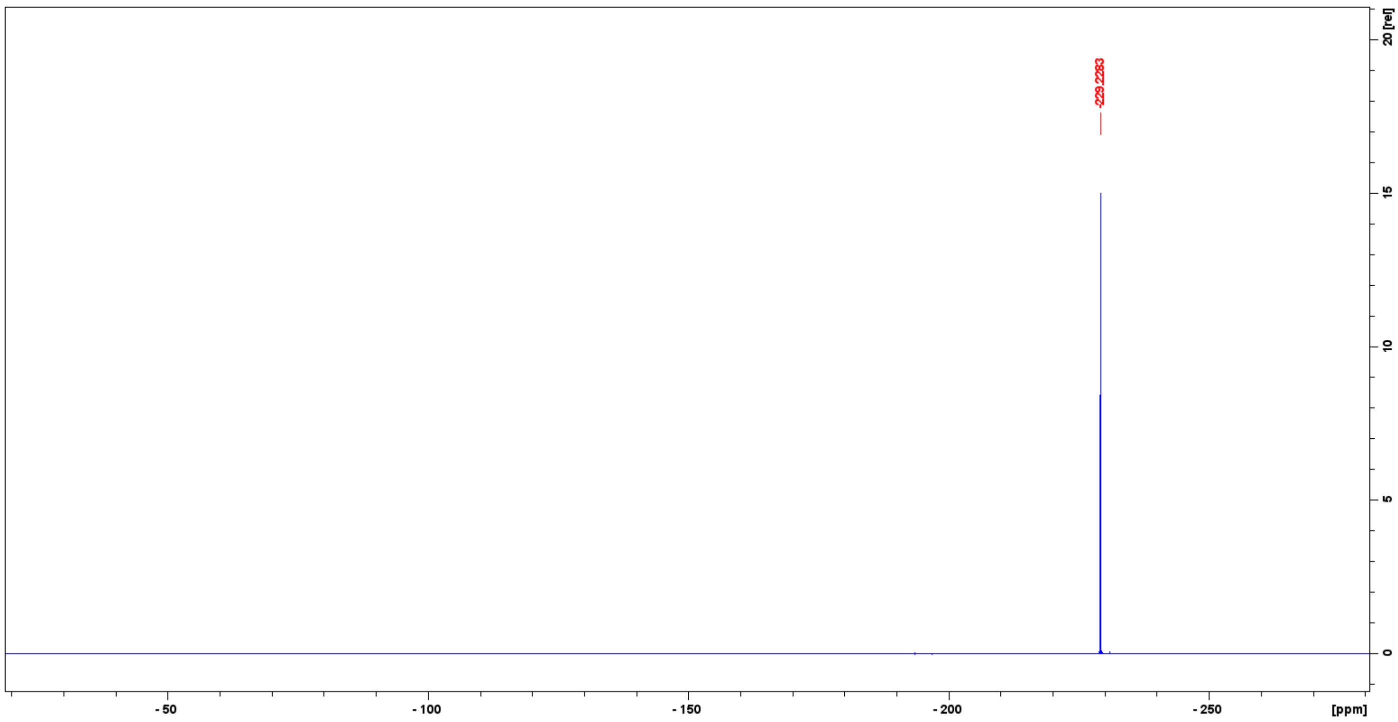

Compound 7 - ^{1}H NMR ($CDCl_3$, 400 MHz)

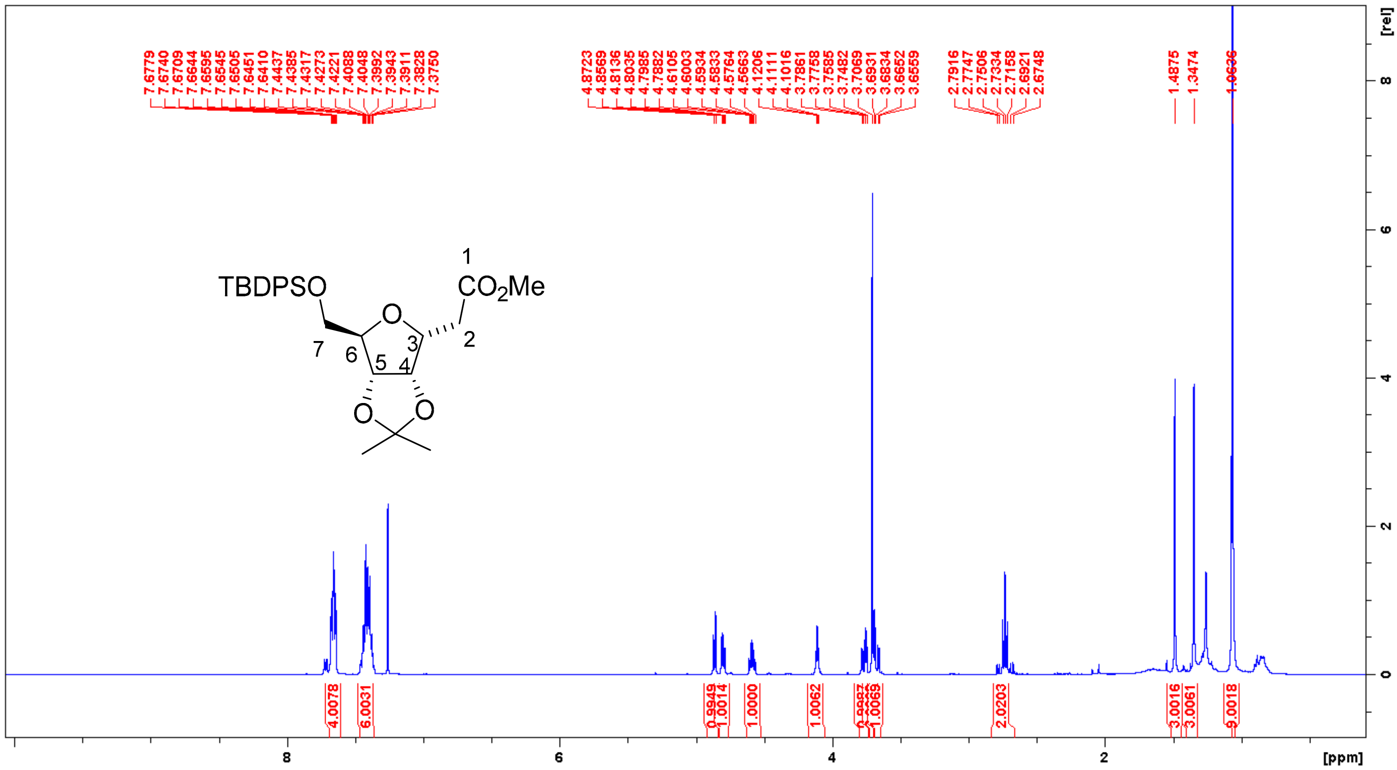

Compound 7 - ^{13}C NMR ($CDCl_3$, 100.6 MHz)

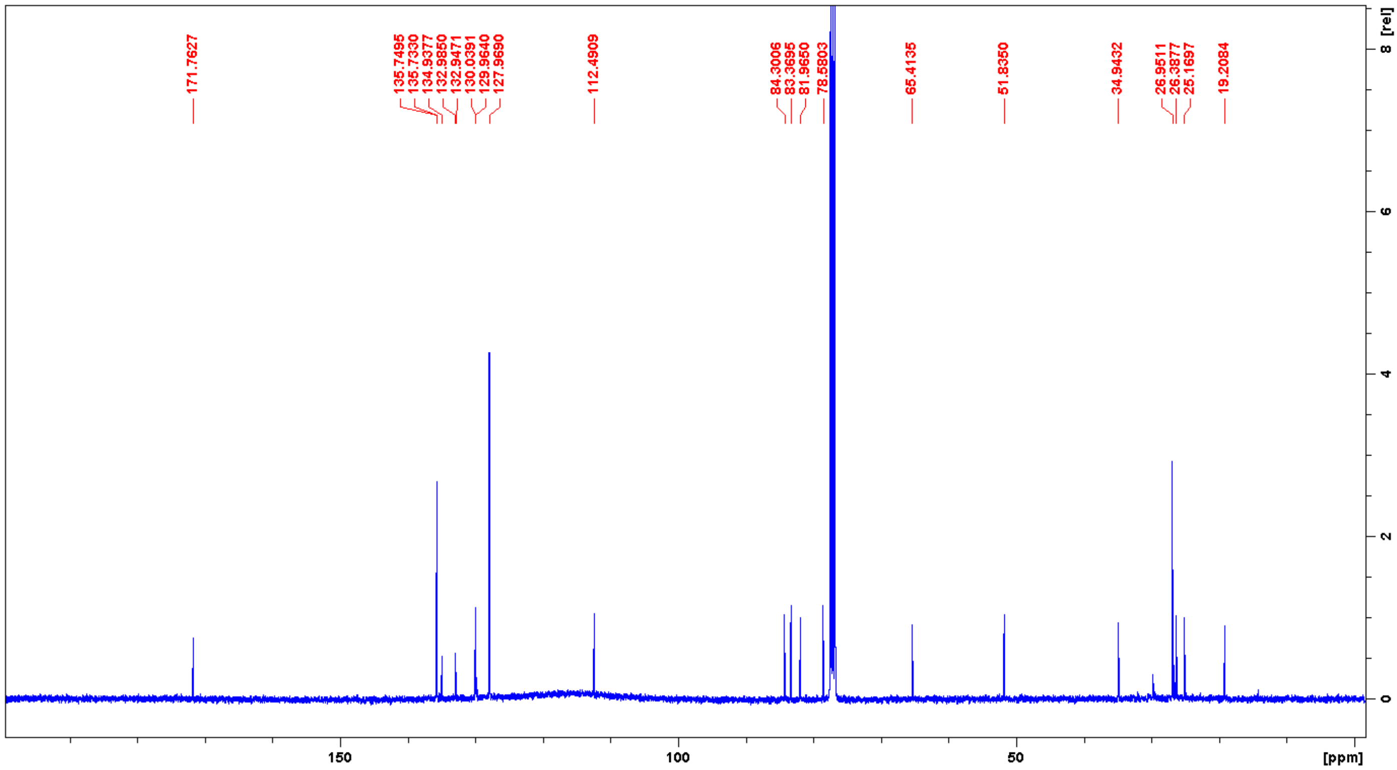

Compound 8 - ^{1}H NMR ($CDCl_3$, 400 MHz)

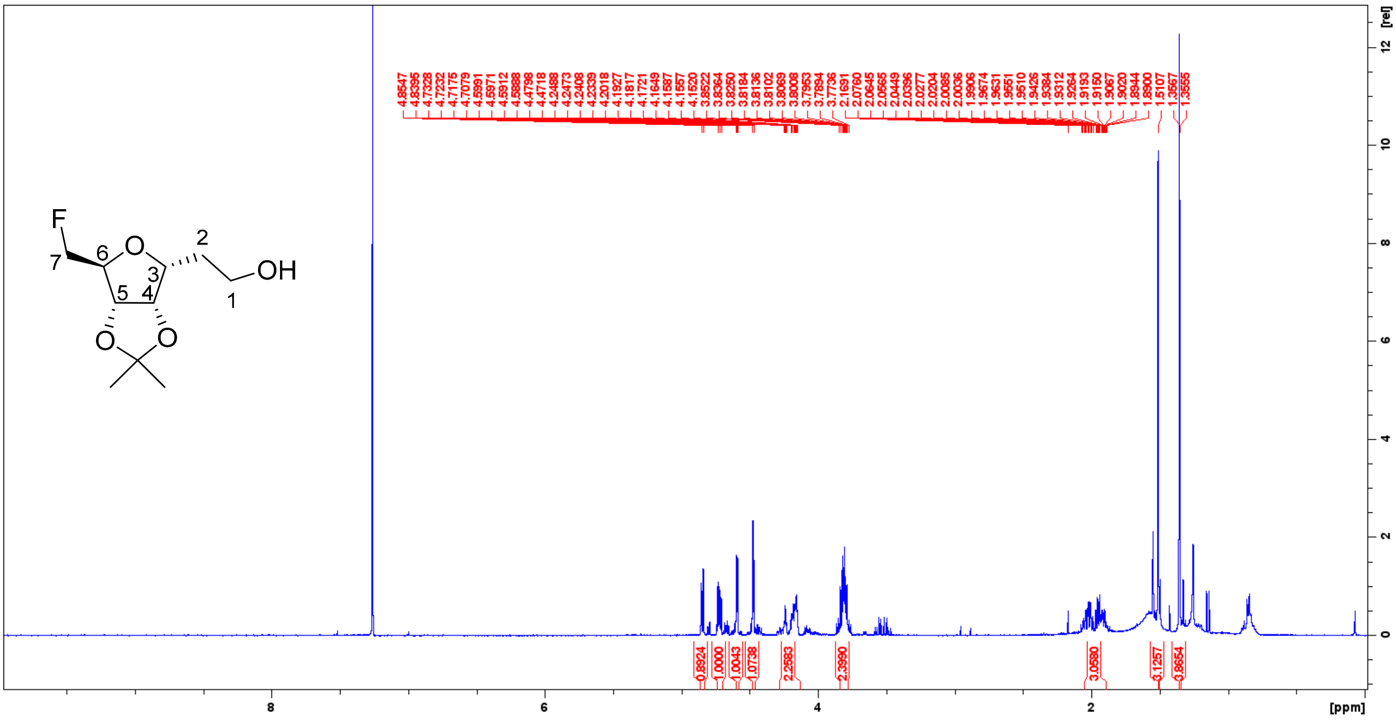

Compound 8 – ^{13}C NMR ($CDCl_3$, 100.6 MHz)

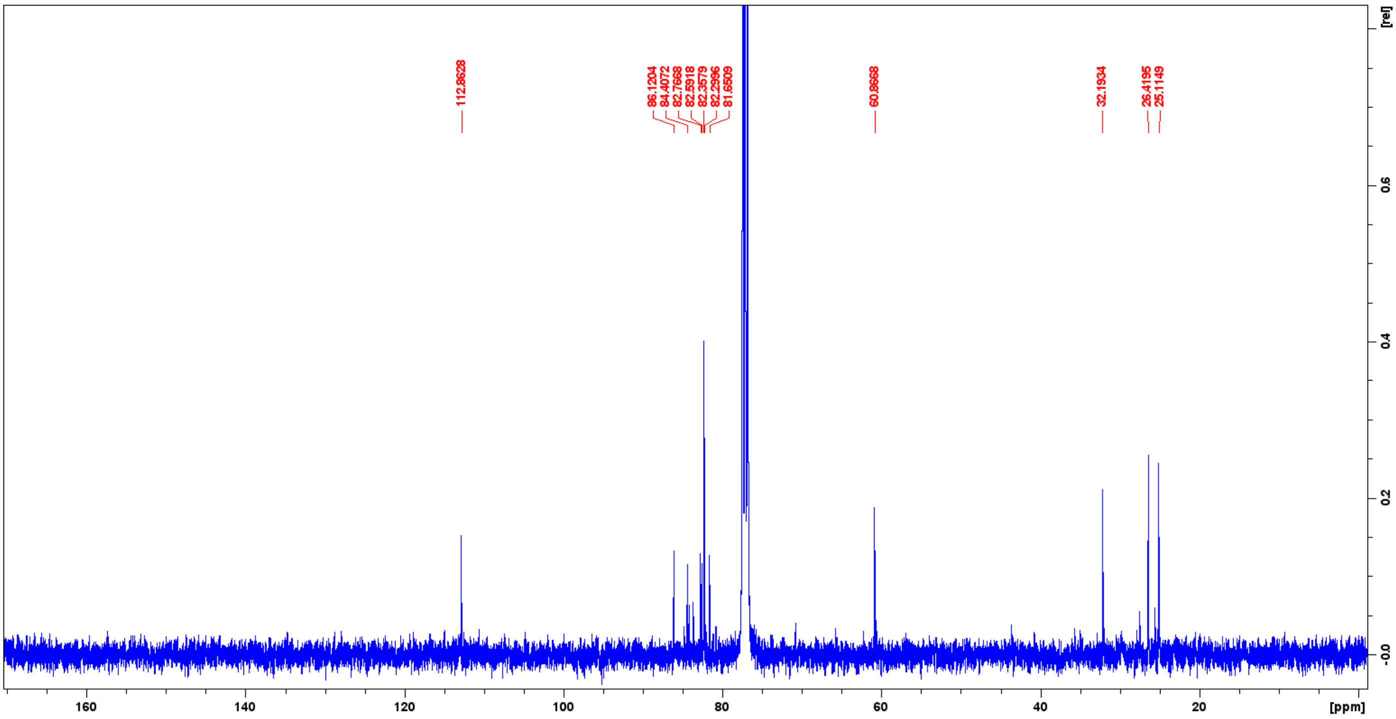

Compound 8 – ^{19}F NMR ($CDCl_3$, 376 MHz)

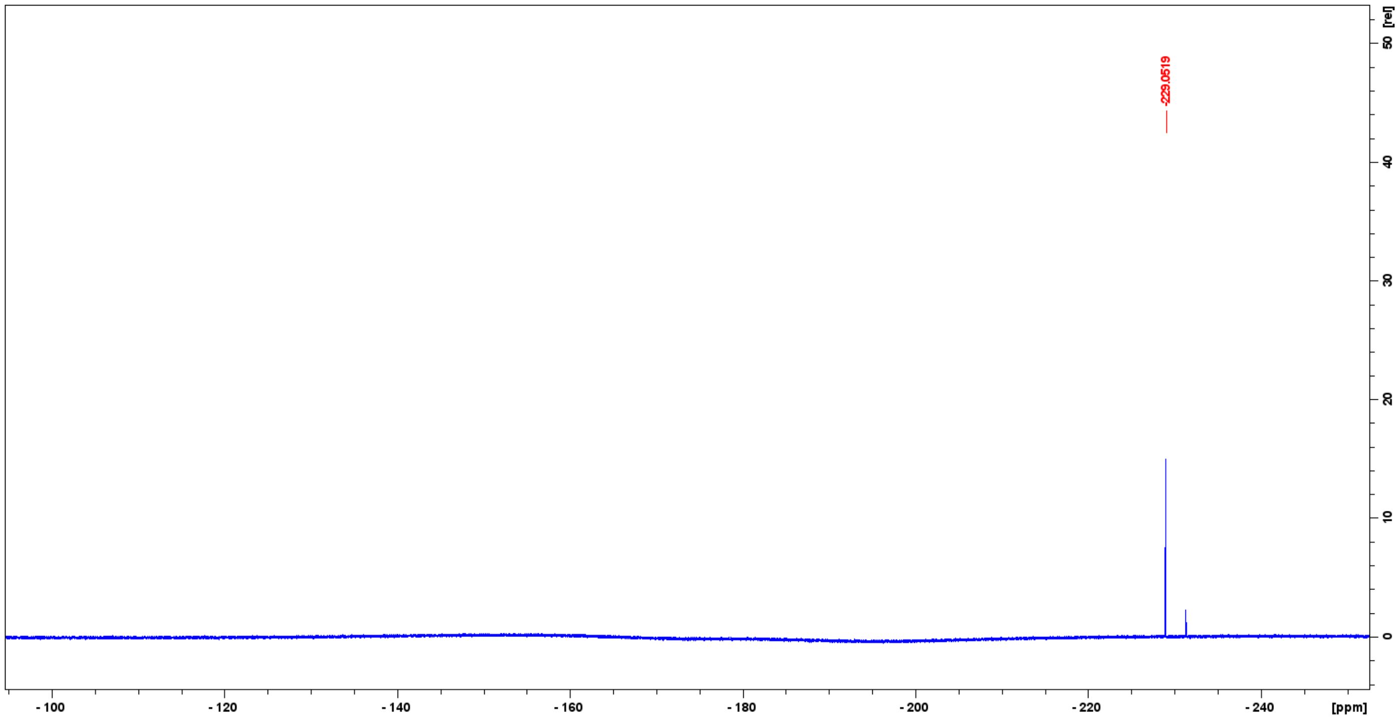

Compound 9 – ^{1}H NMR ($CDCl_3$, 400 MHz)

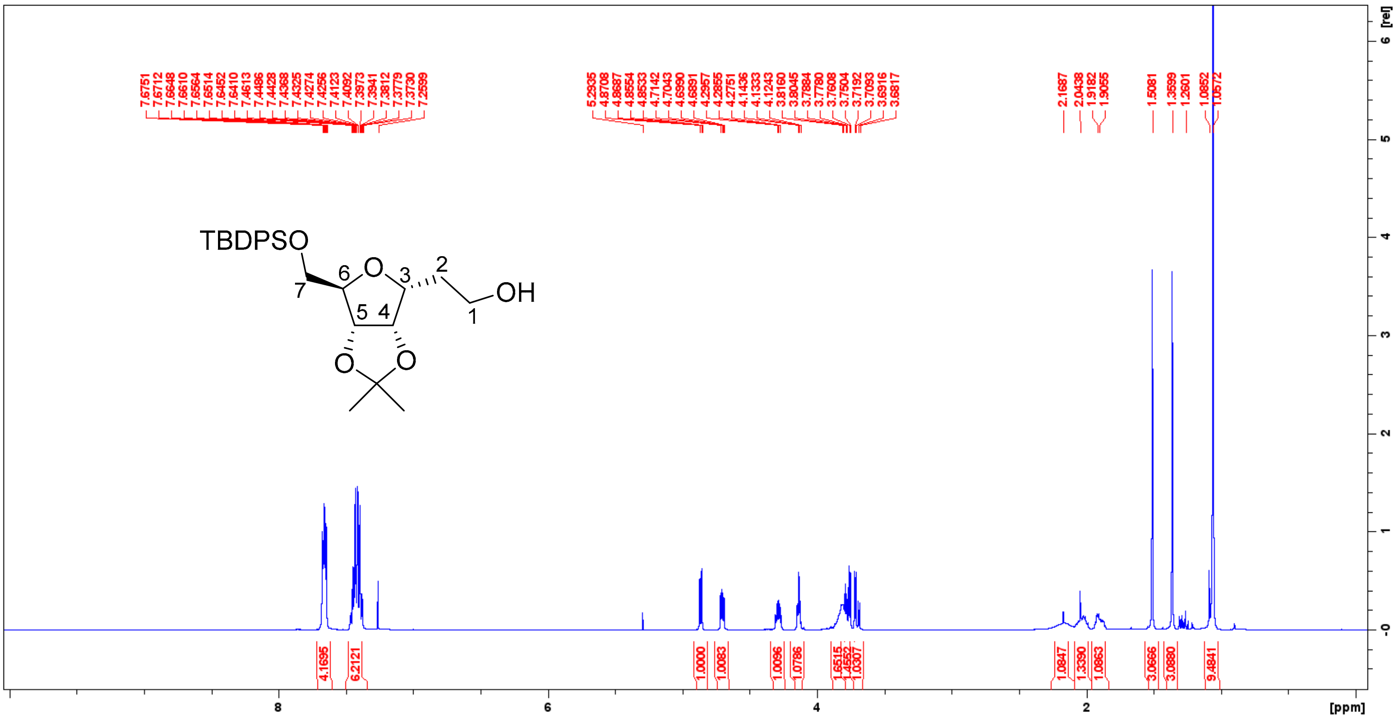

Compound 9 – ^{13}C NMR ($CDCl_3$, 100.6 MHz)

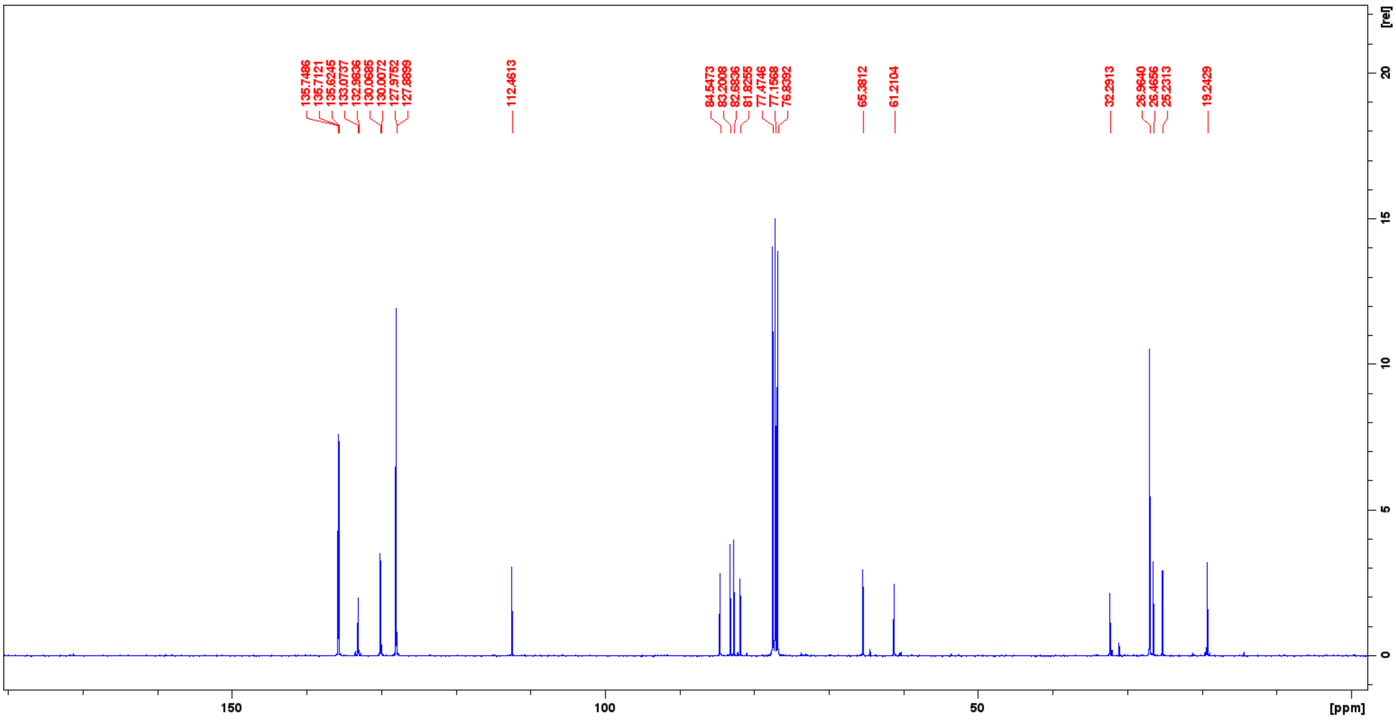

Compound 10 - ^{1}H NMR ($CDCl_3$, 400 MHz)

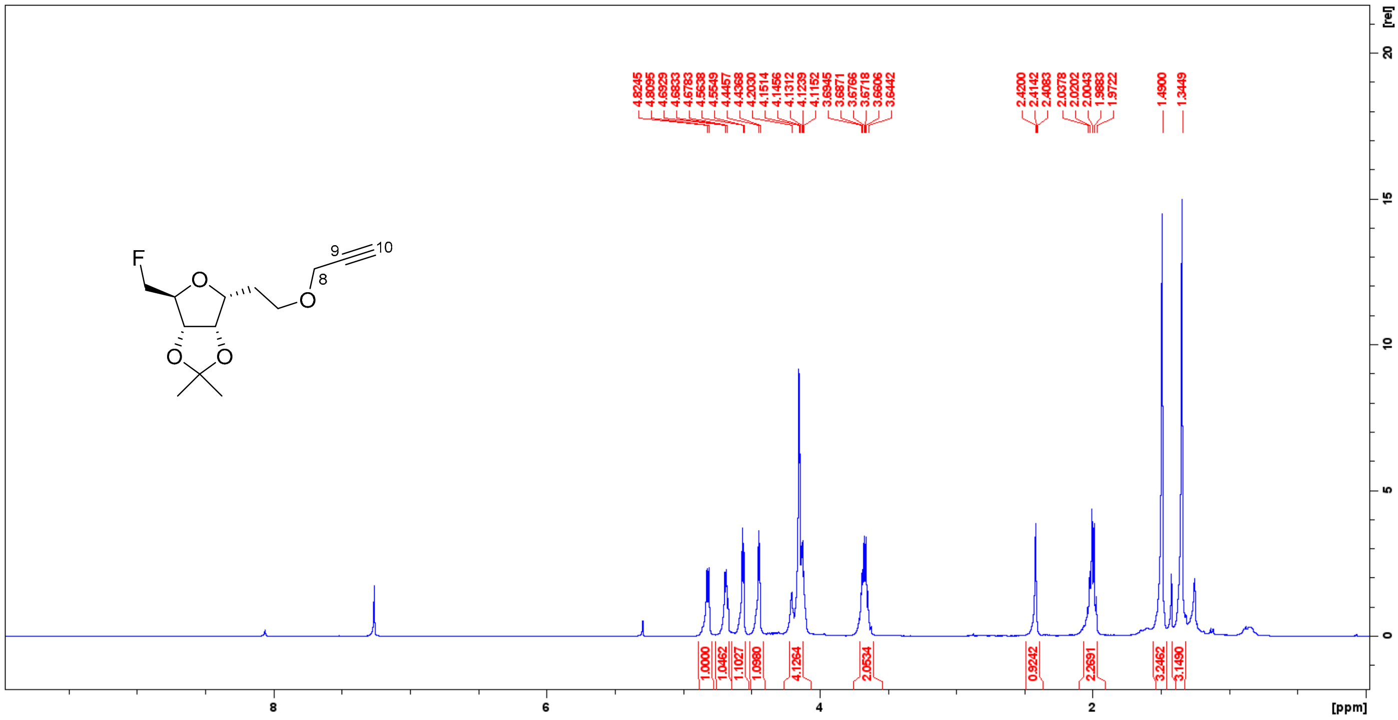

Compound 10 – ^{13}C NMR ($CDCl_3$, 100.6 MHz)

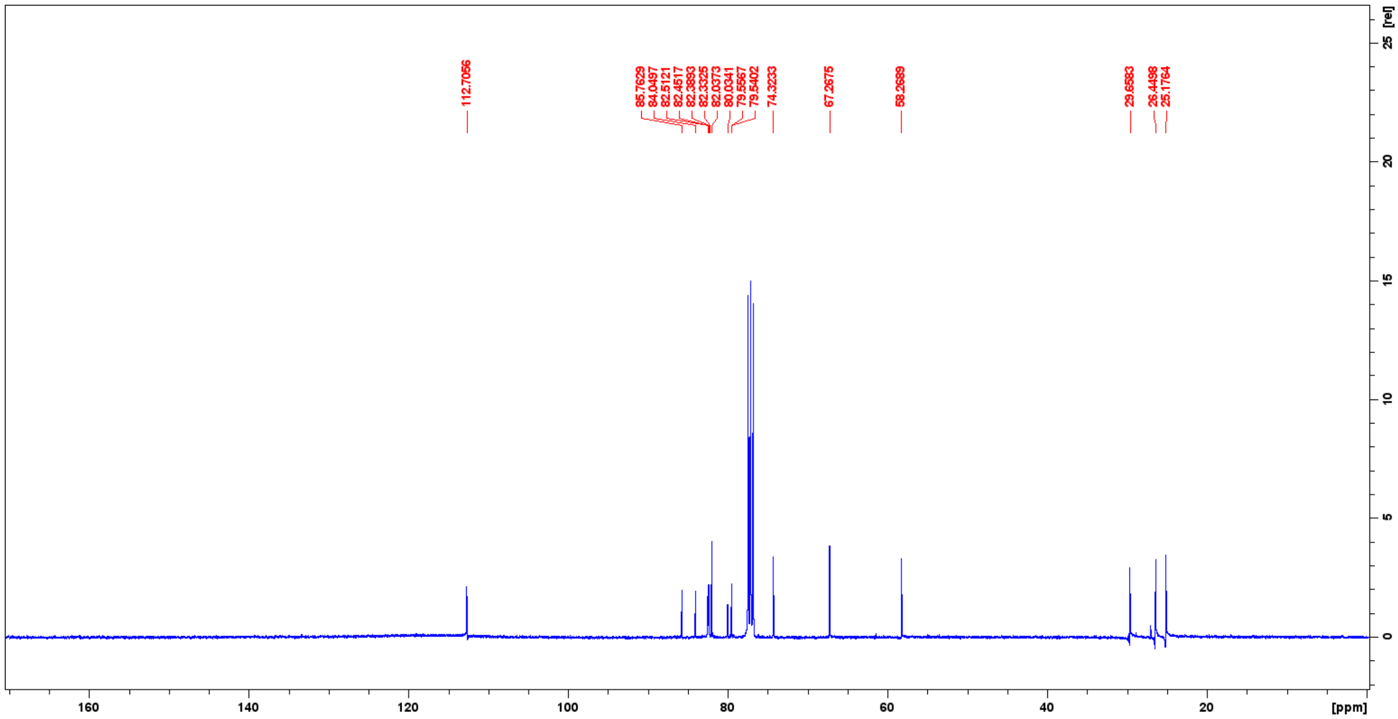

Compound 11 - ^{1}H NMR ($CDCl_3$, 400 MHz)

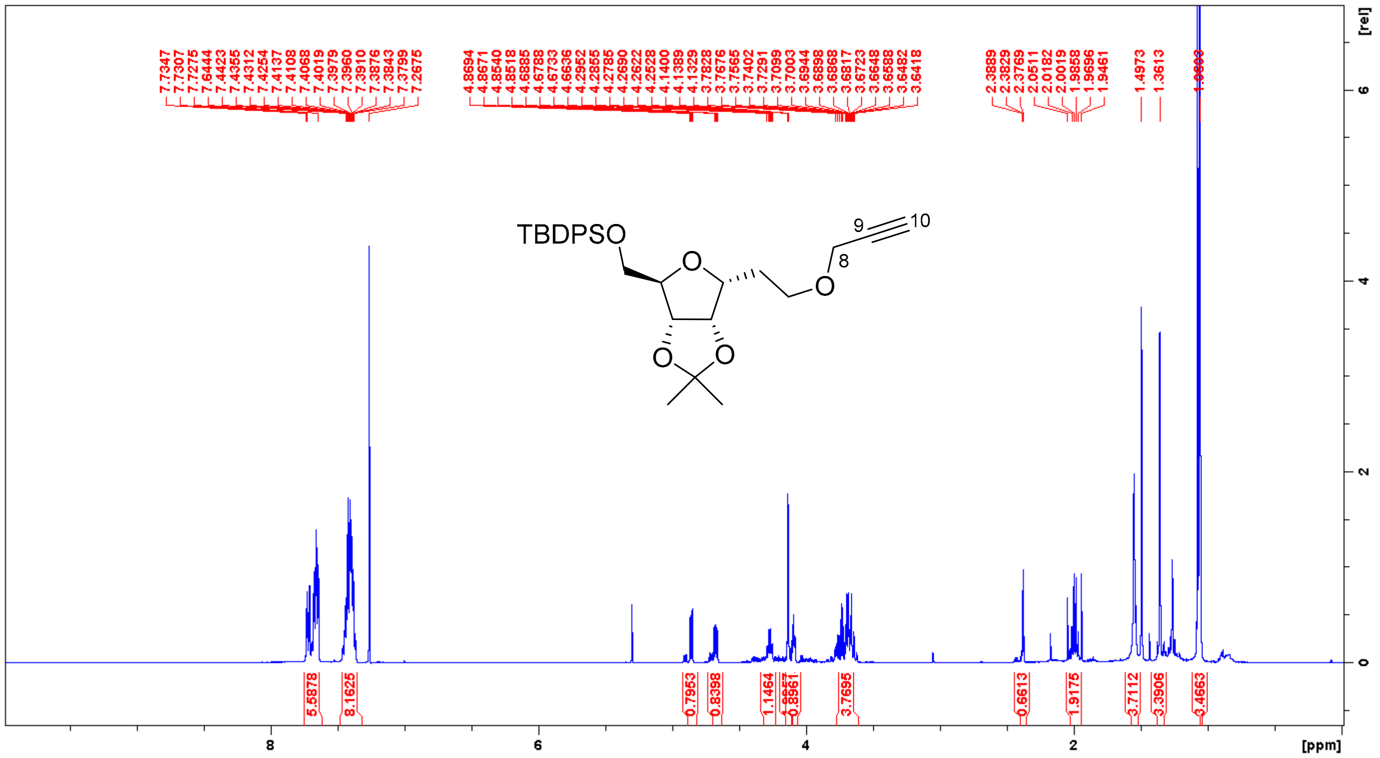

Compound 11 - ^{13}C NMR ($CDCl_3$, 100.6 MHz)

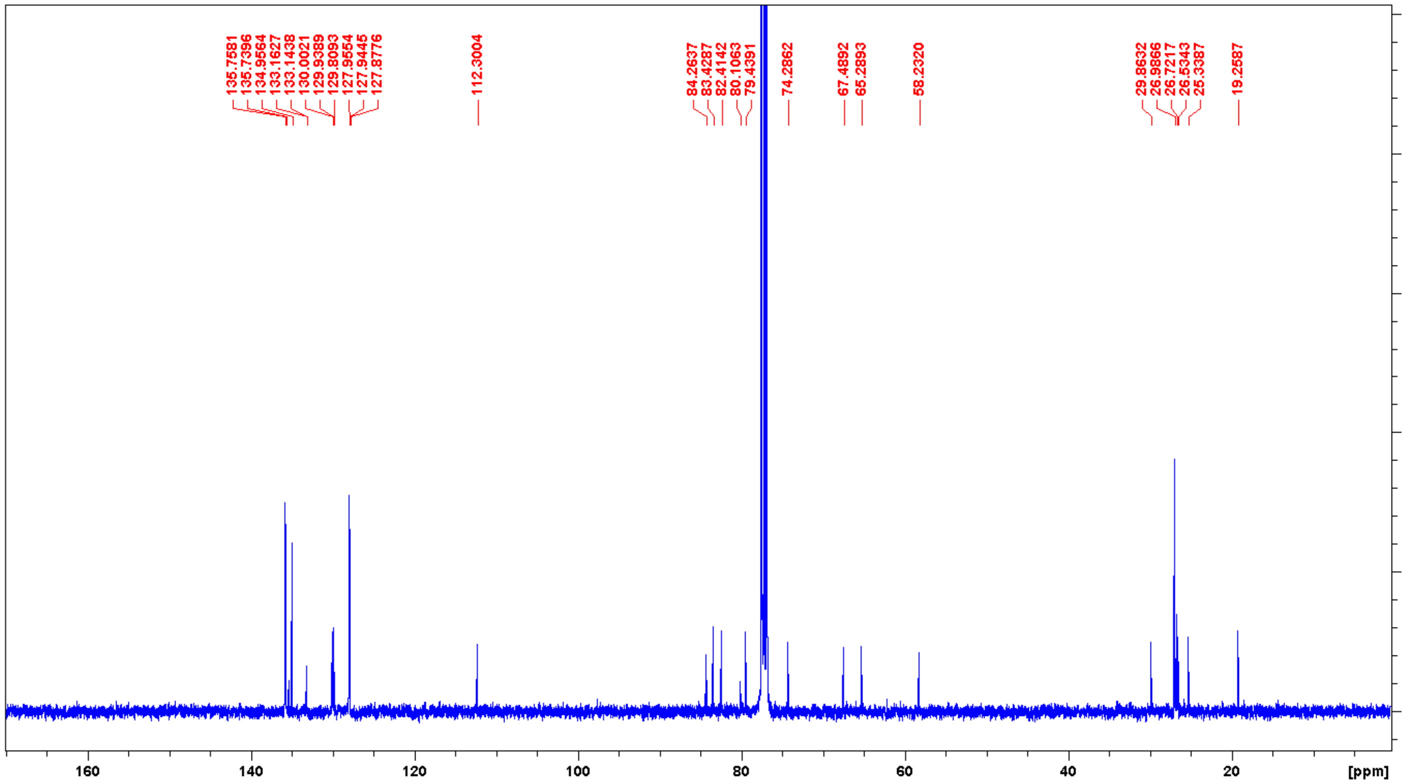

Compound 12 - ^{1}H NMR ($CDCl_3$, 400 MHz)

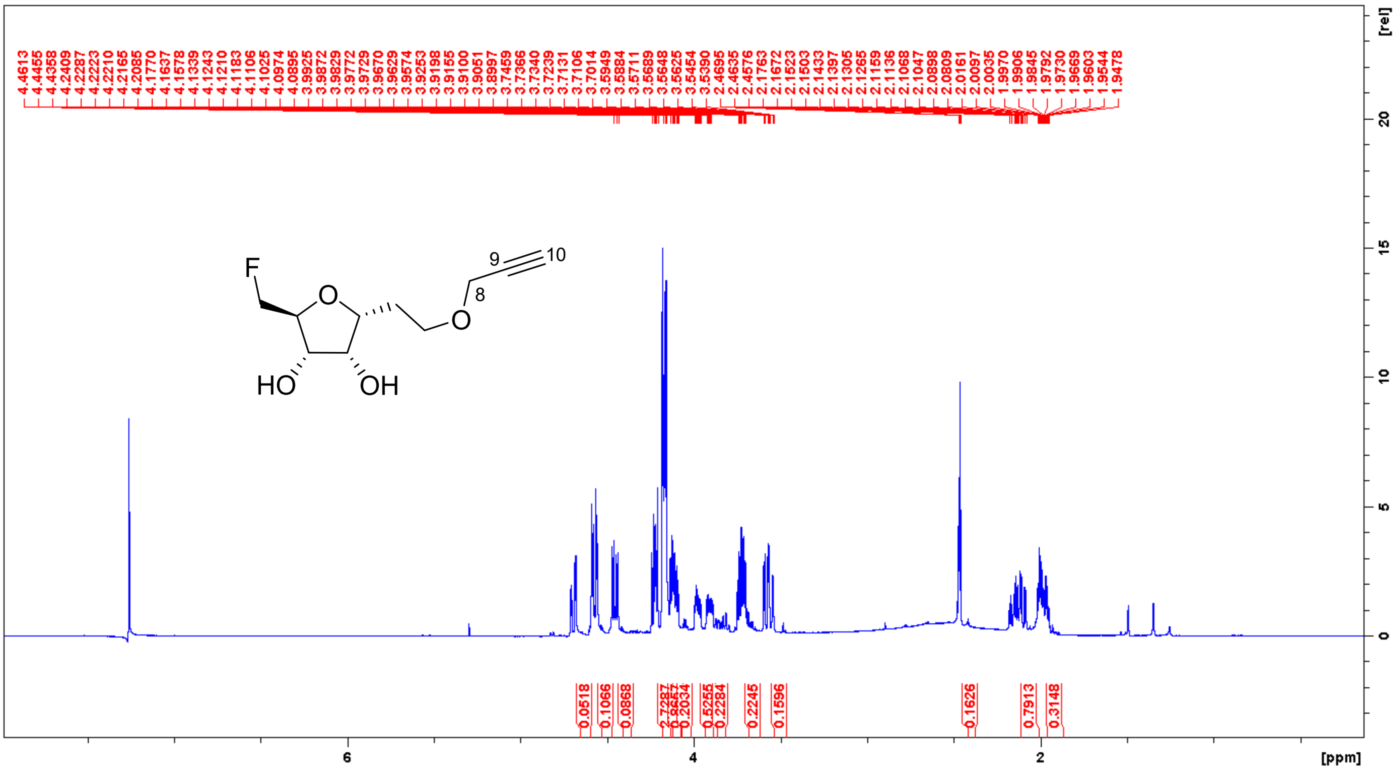

Compound 12 - ^{13}C NMR ($CDCl_3$, 100.6 MHz)

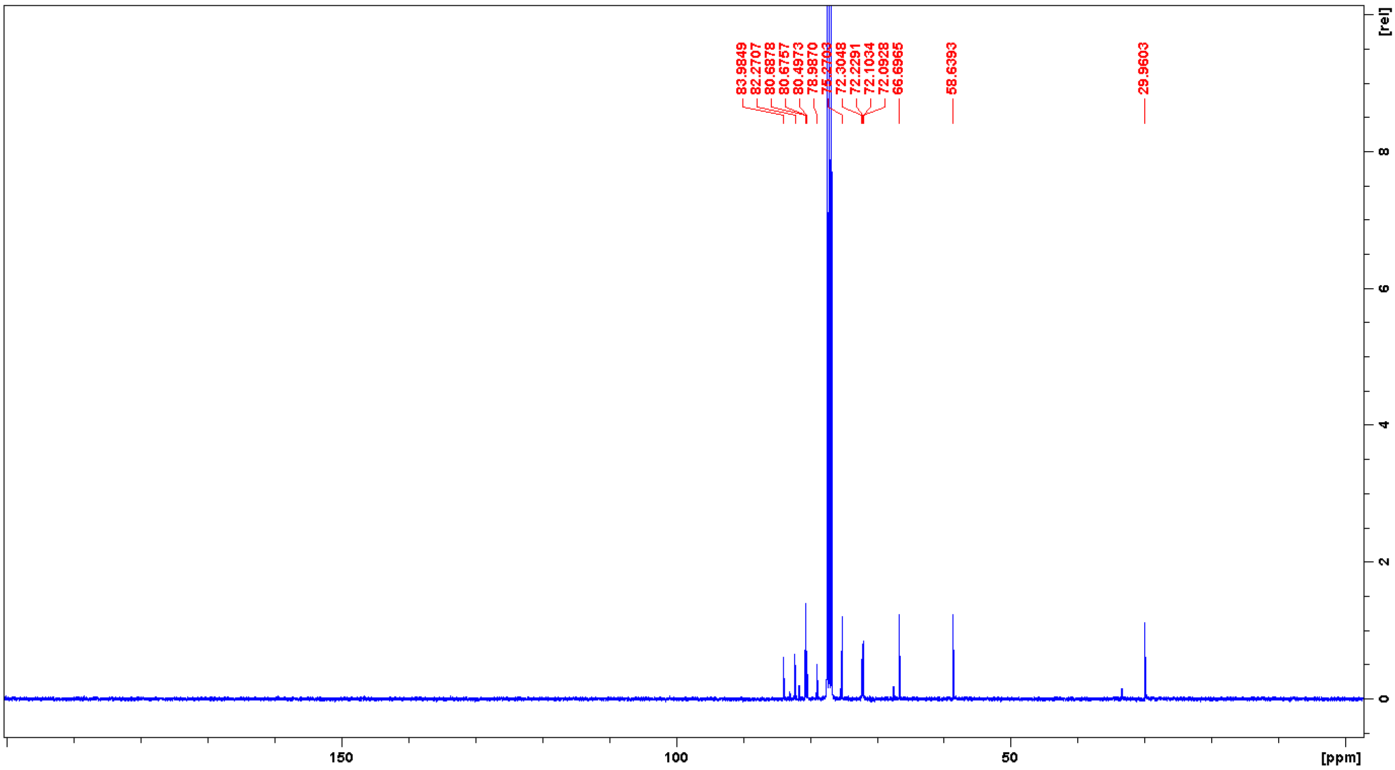

Compound 12 – ^{19}F NMR ($CDCl_3$, 376 MHz)

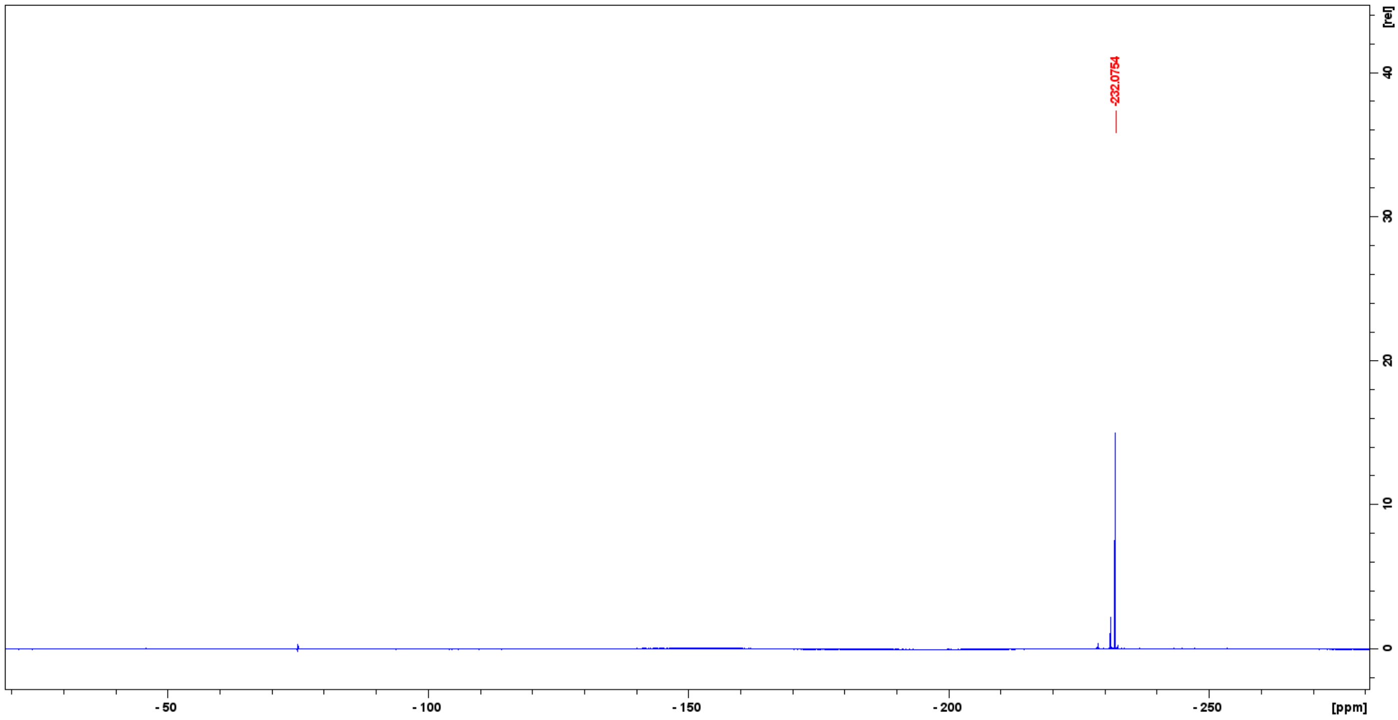

Compound 13 - ^{1}H NMR ($CDCl_3$, 400 MHz)

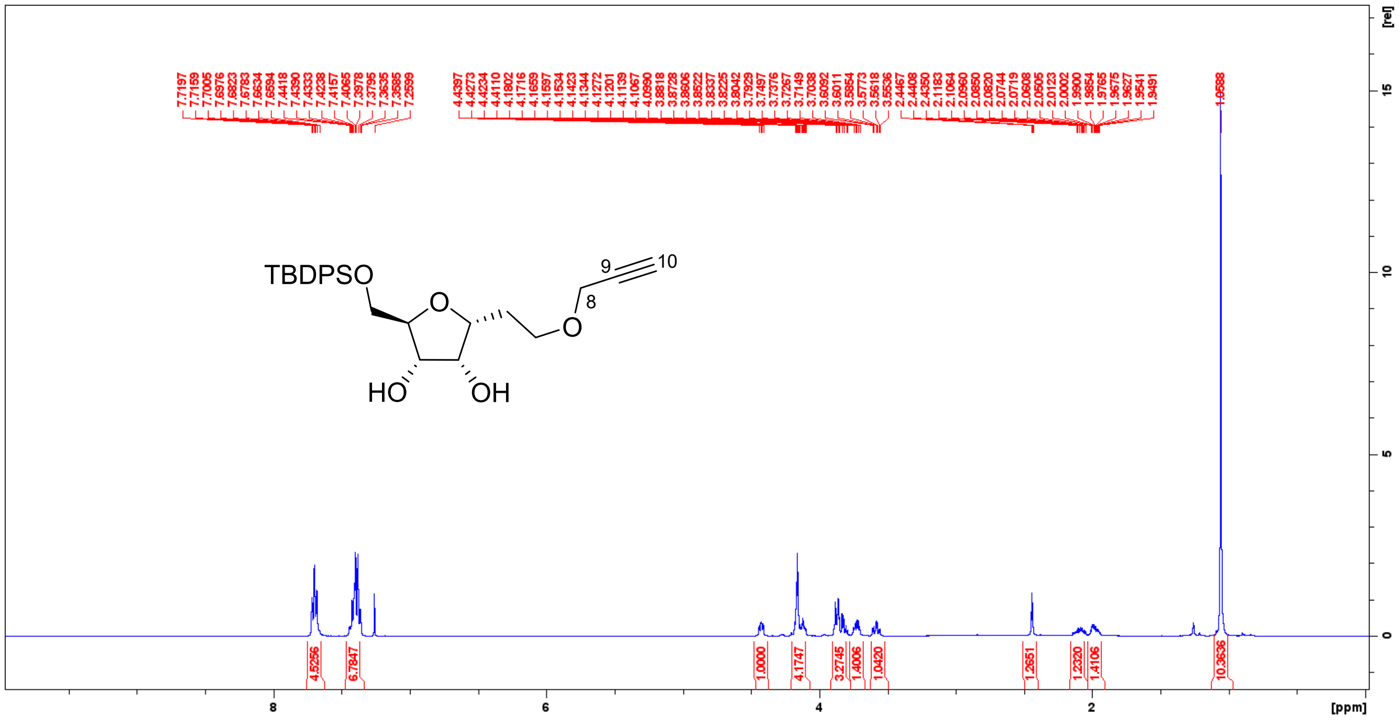

Compound 13 - ^{13}C NMR ($CDCl_3$, 100.6 MHz)

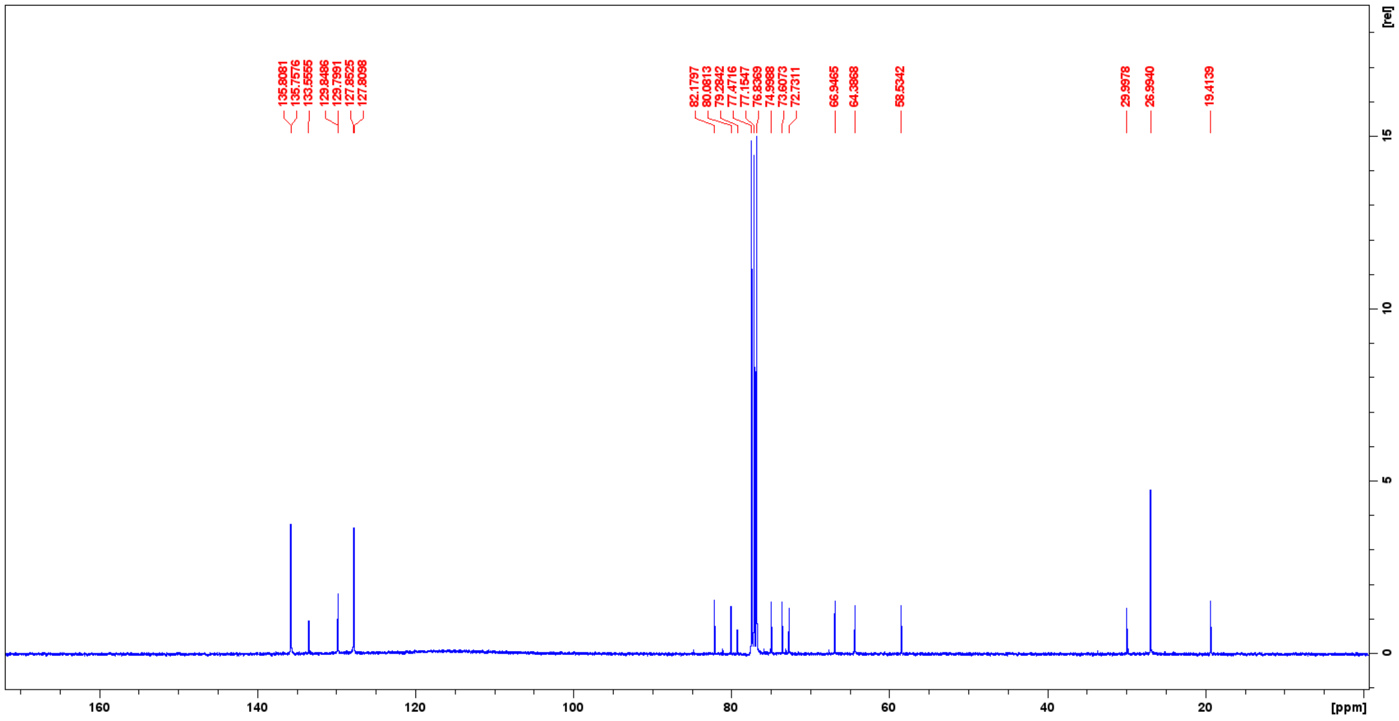

Compound 14 - ^{1}H NMR ($CDCl_3$, 400 MHz)

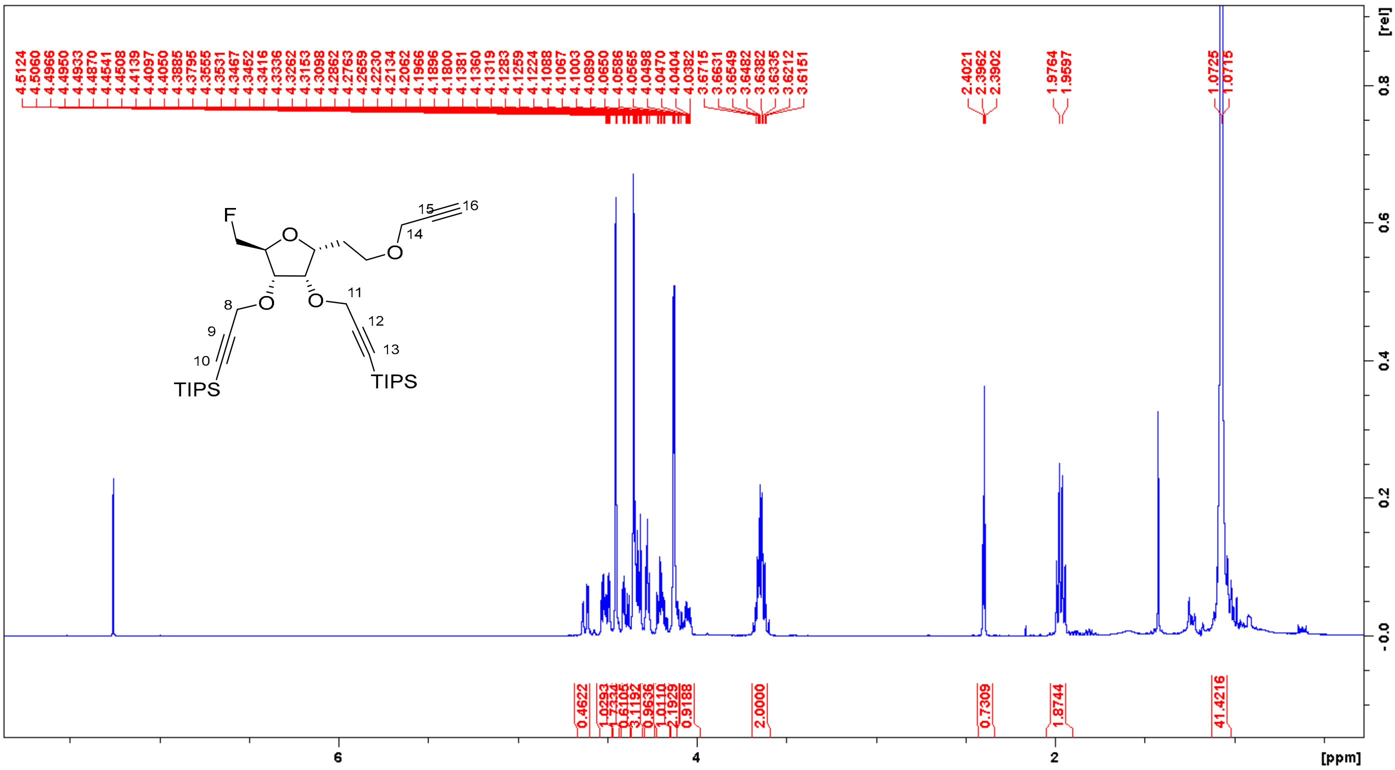

Compound 14 - ^{13}C NMR ($CDCl_3$, 100.6 MHz)

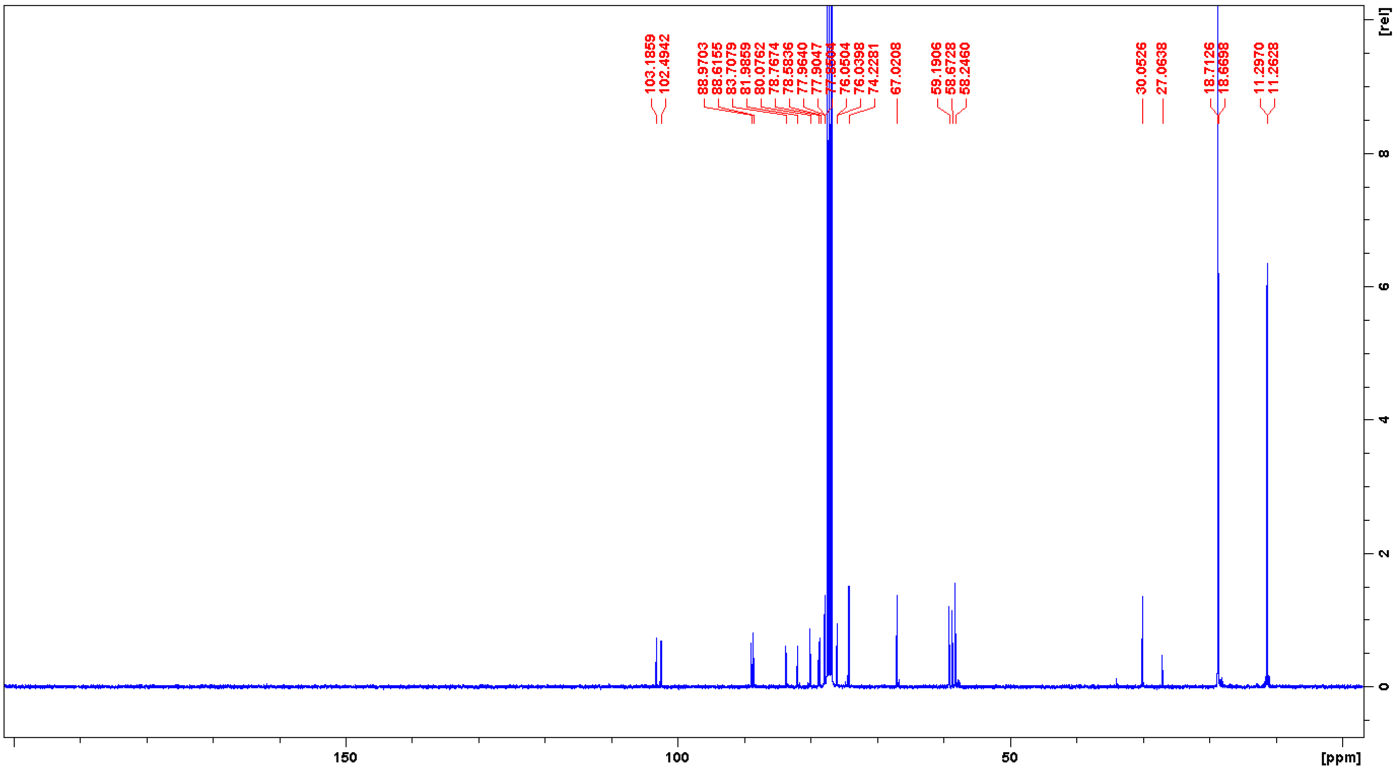

Compound 14 – ^{19}F NMR ($CDCl_3$, 376 MHz)

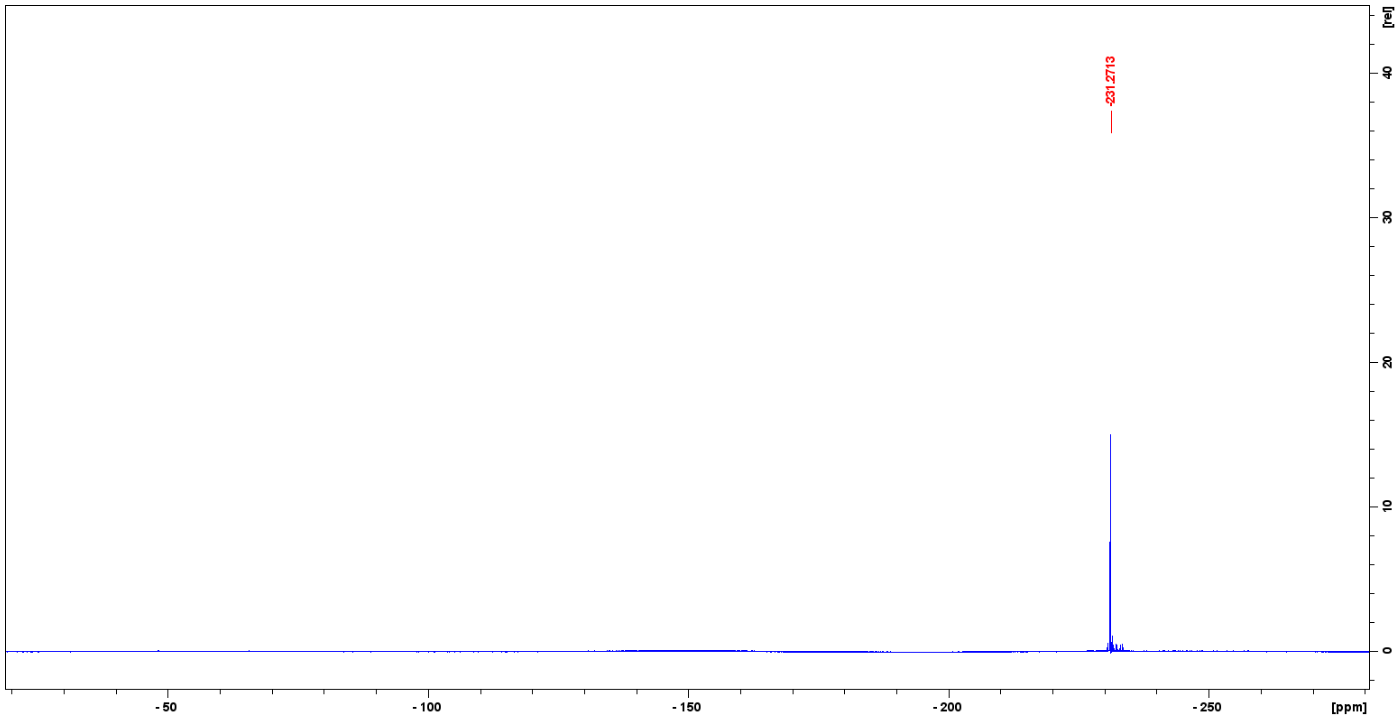

Compound 15 - ^{1}H NMR ($CDCl_3$, 400 MHz)

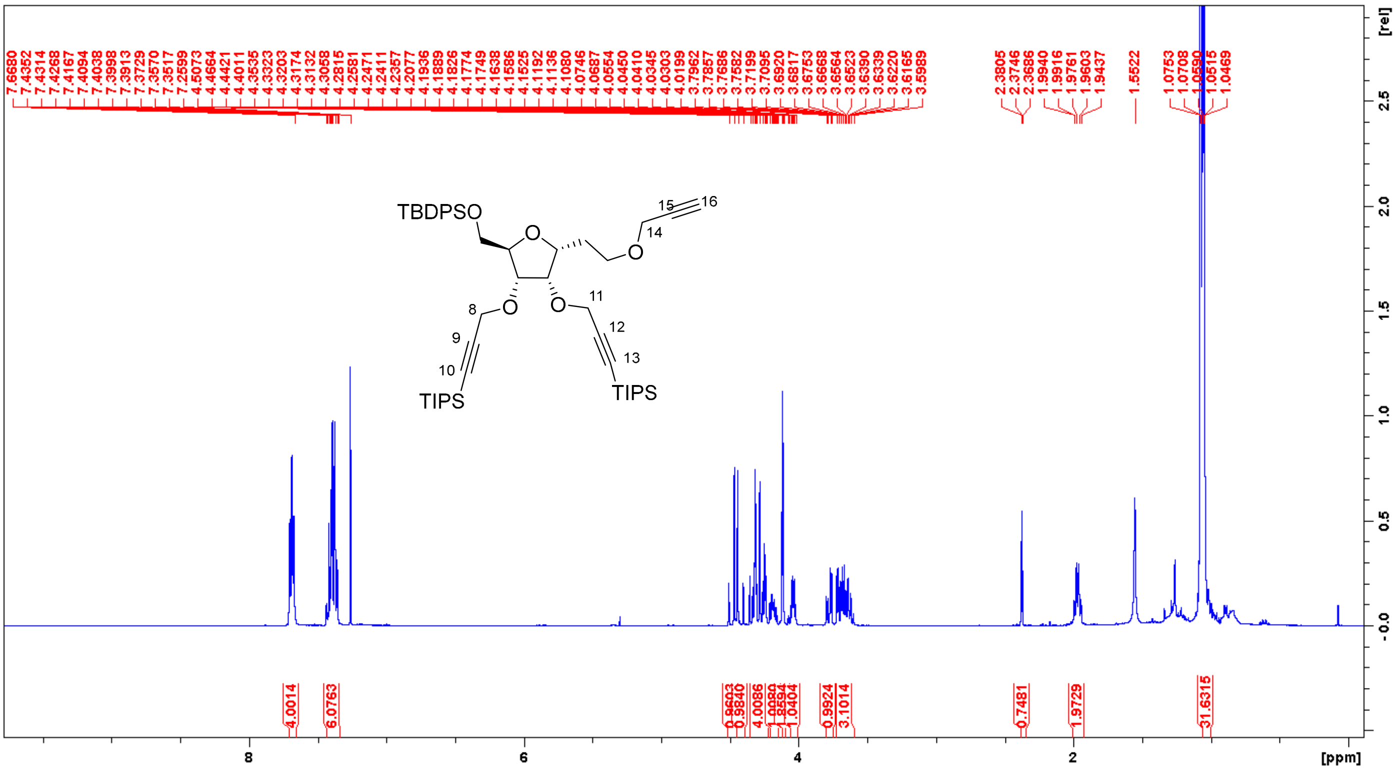

Compound 15 - ^{13}C NMR ($CDCl_3$, 100.6 MHz)

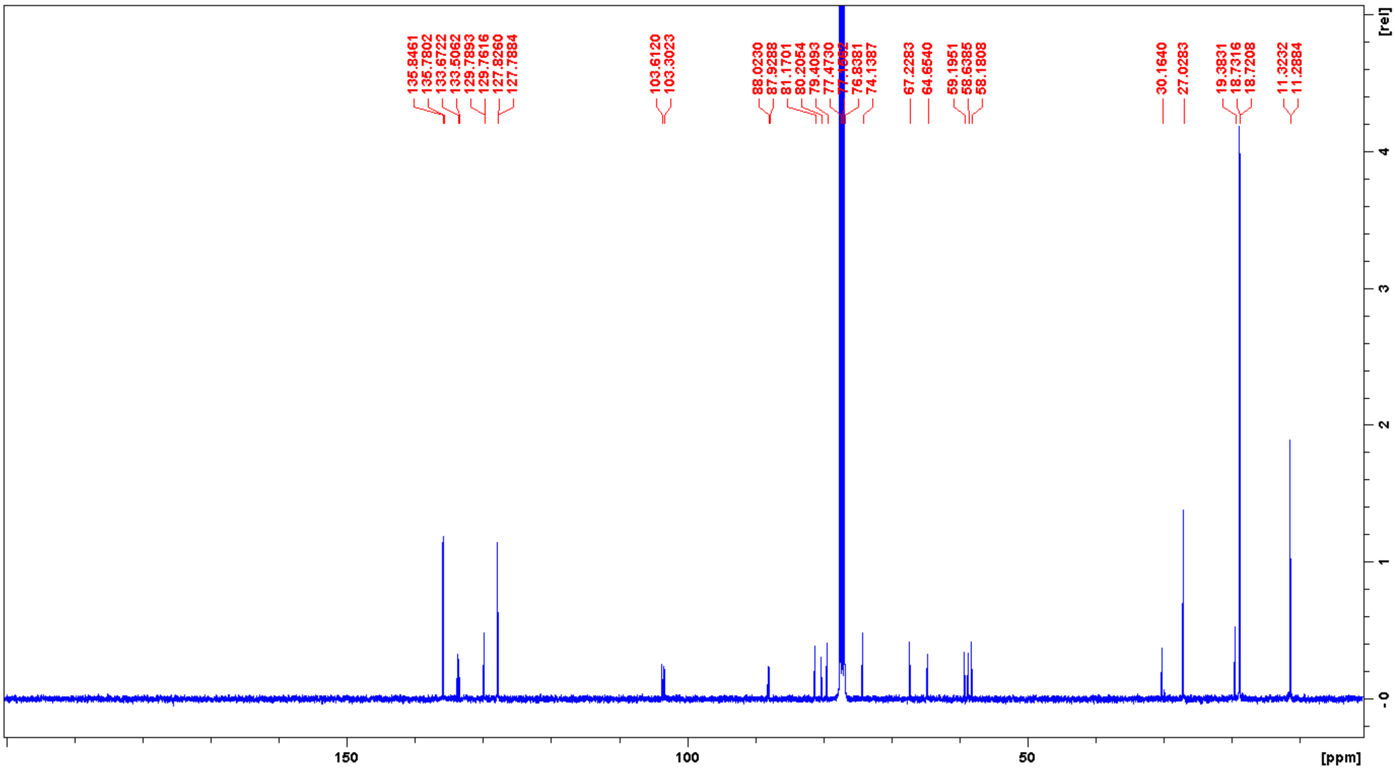

Compound 16 - ^{1}H NMR ($CDCl_3$, 400 MHz)

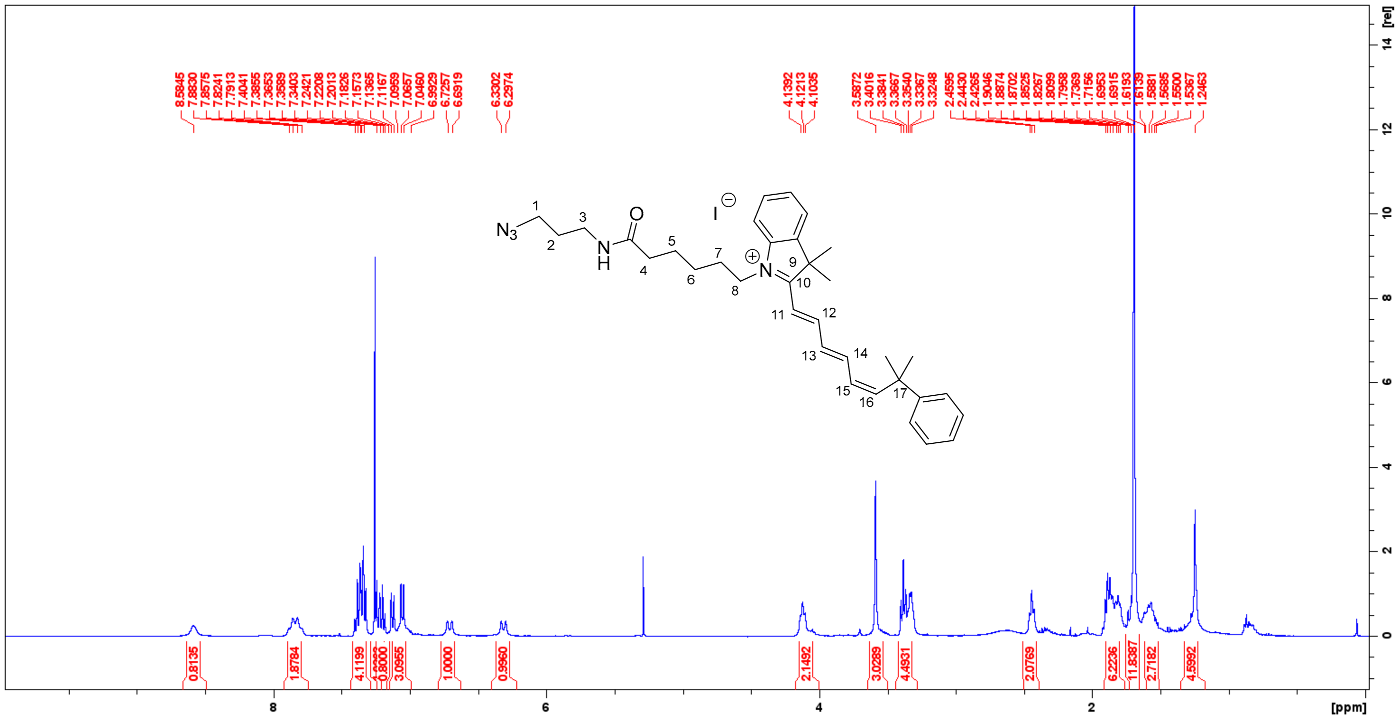

Compound 16 – ^{13}C NMR ($CDCl_3$, 100.6 MHz)

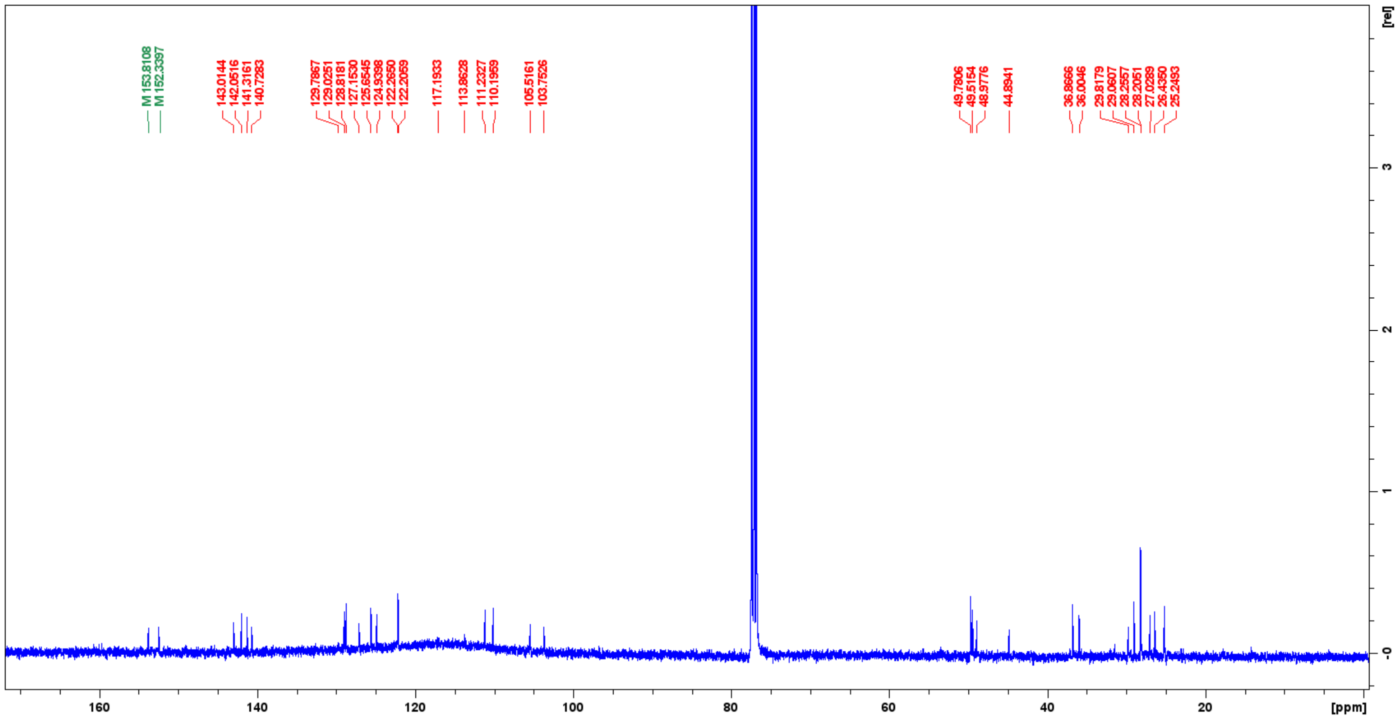

Compound 17 - ^{1}H NMR ($CDCl_3$, 400 MHz)

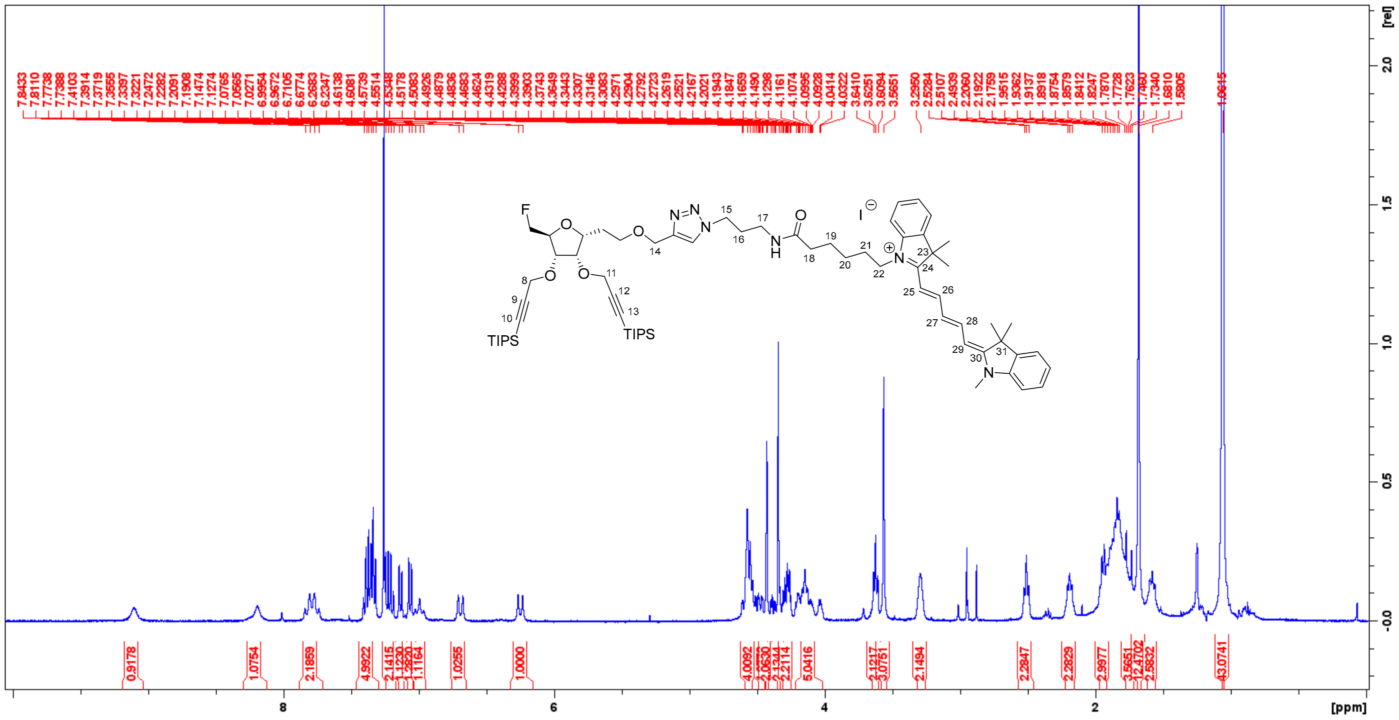

Compound 17 – ^{13}C NMR ($CDCl_3$, 100.6 MHz)

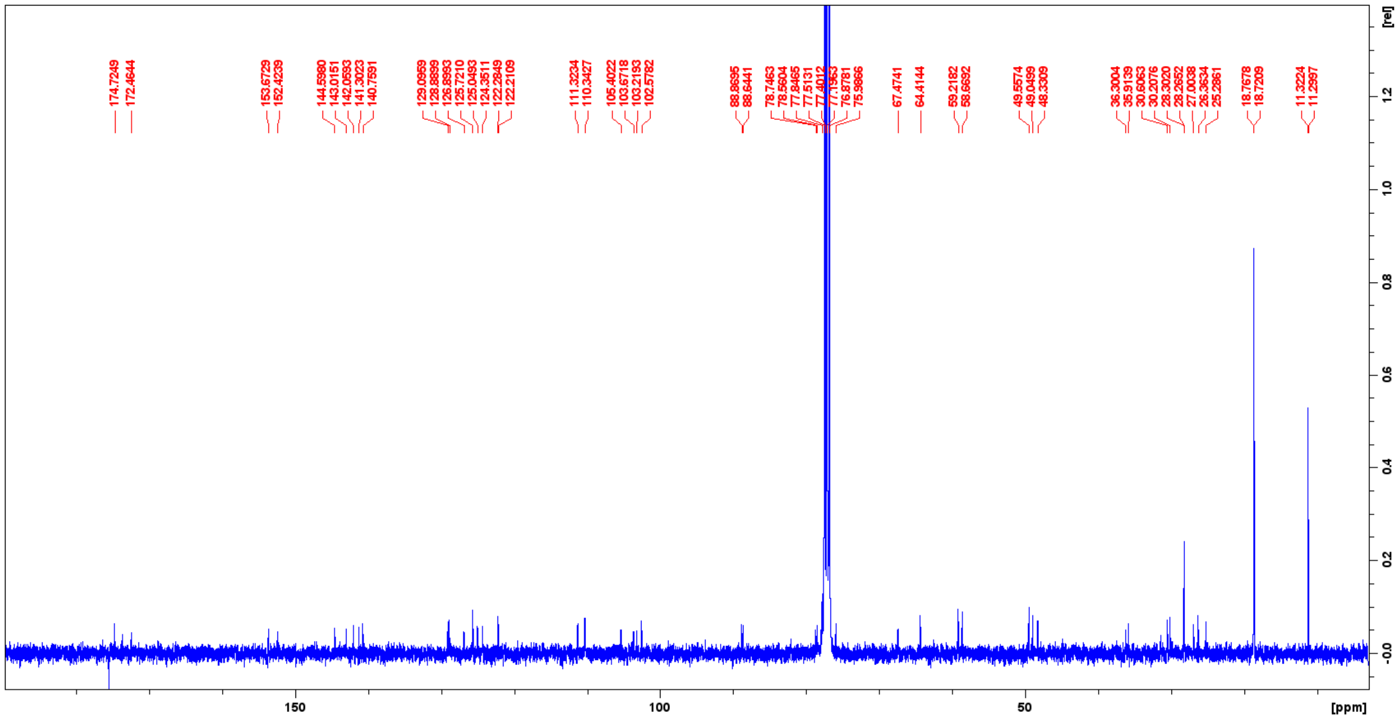

Compound 17 – ^{19}F NMR ($CDCl_3$, 376 MHz)

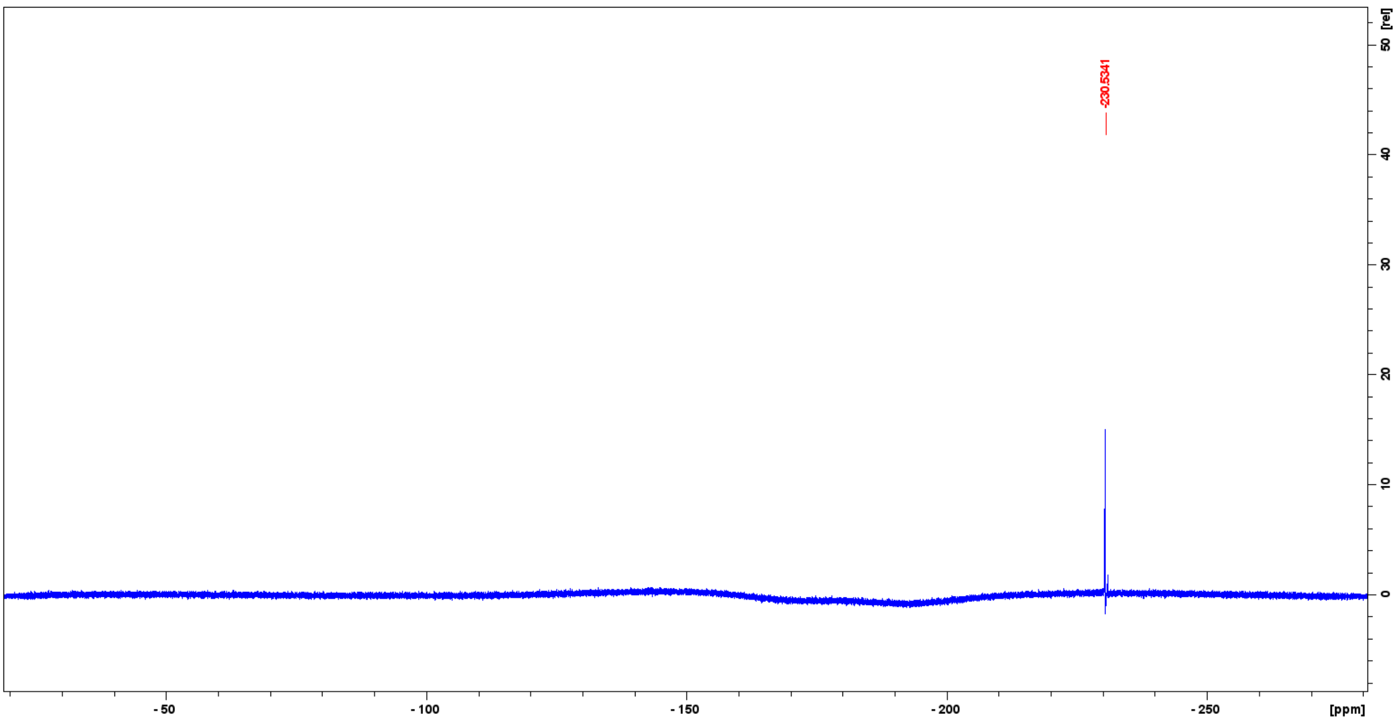

Compound 18 - ^{1}H NMR ($CDCl_3$, 400 MHz)

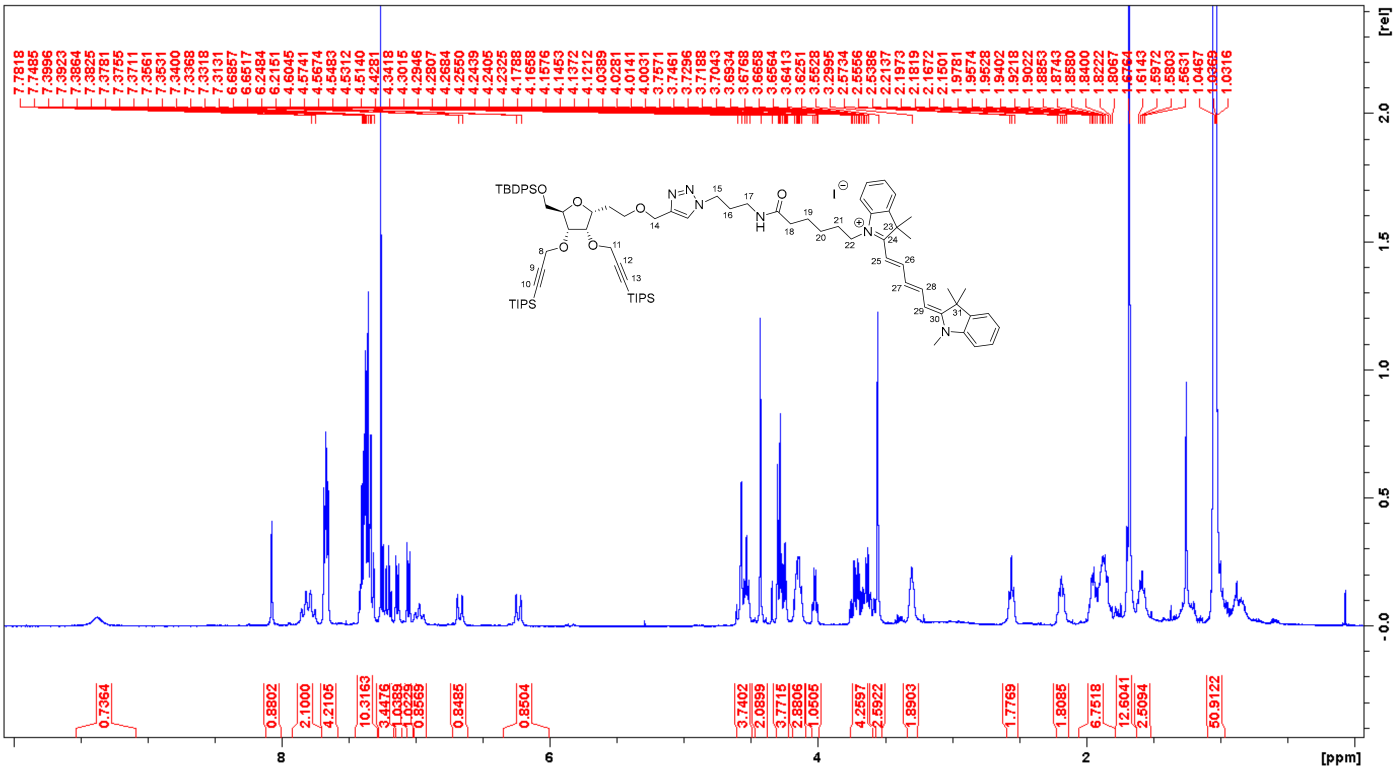

Compound 18 - ^{13}C NMR ($CDCl_3$, 100.6 MHz)

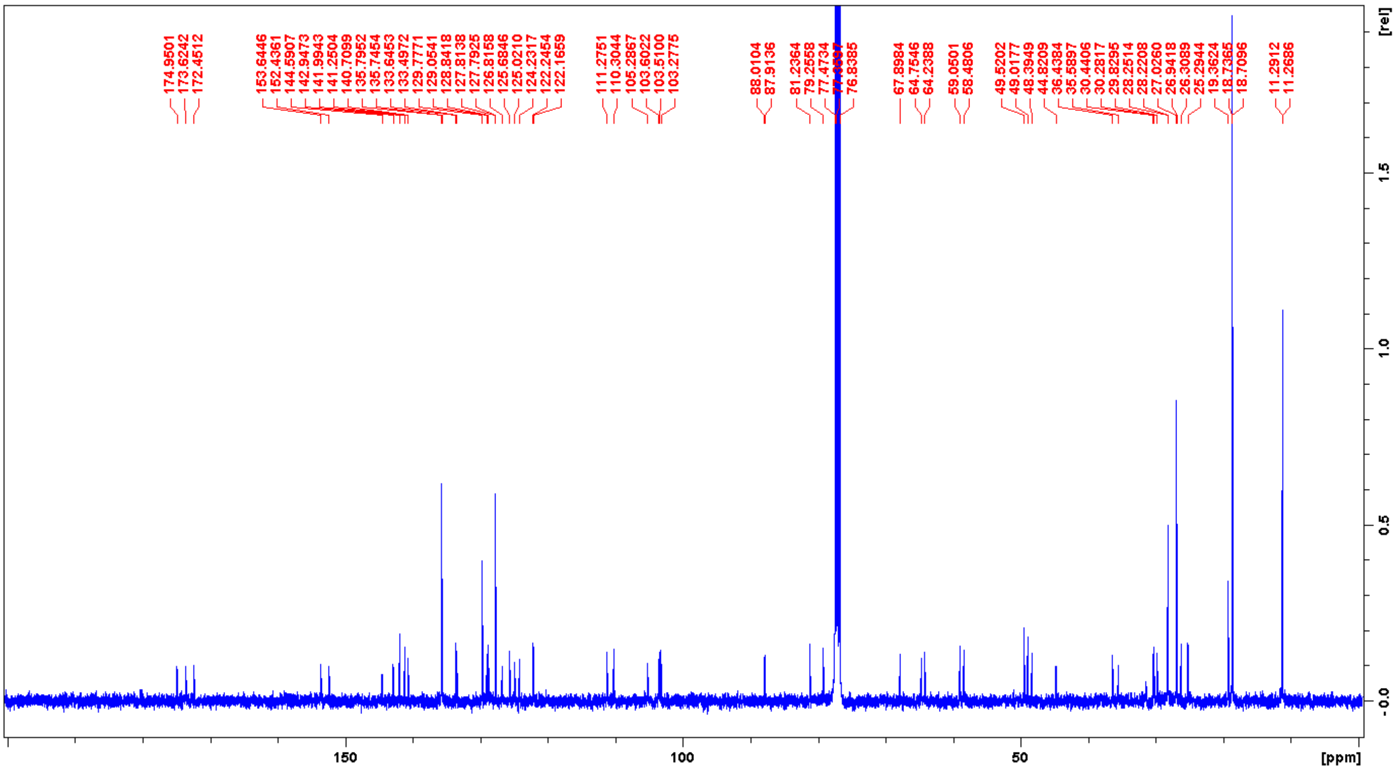

Compound 19 - ^{1}H NMR ($CDCl_3$, 400 MHz)

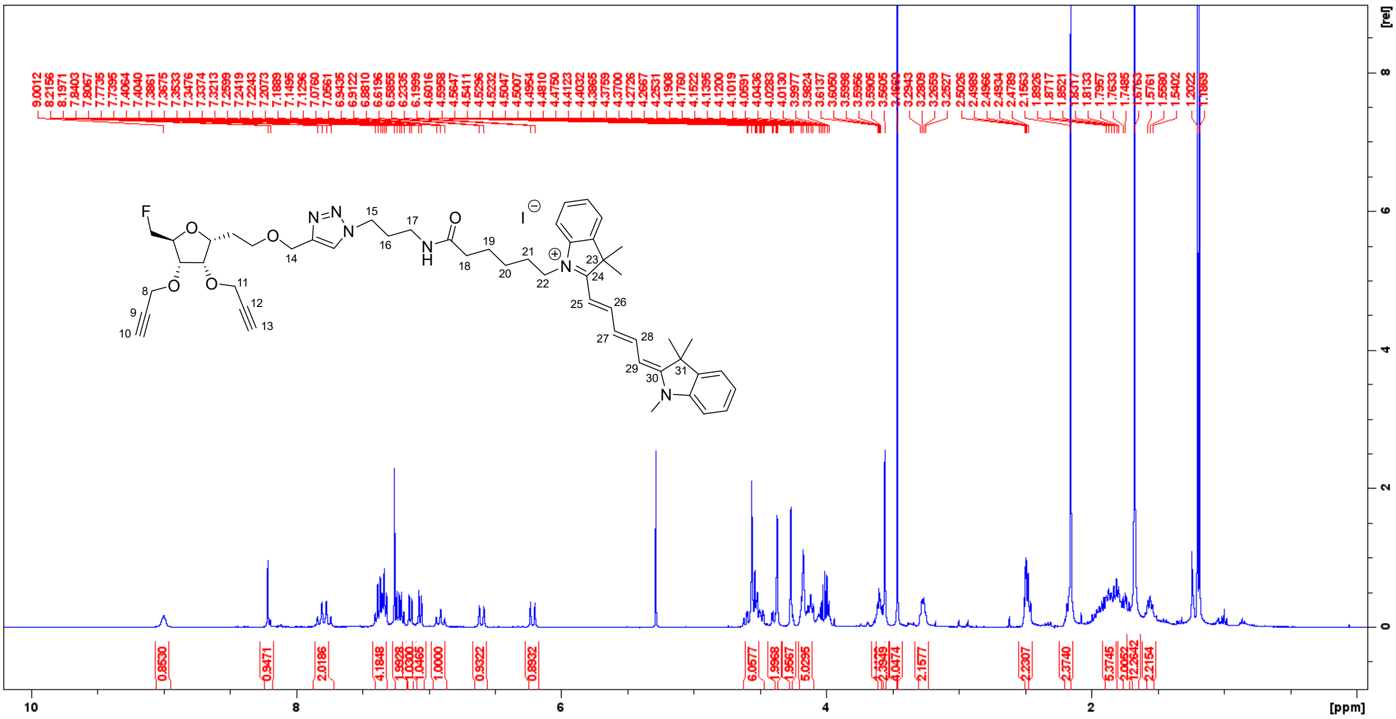

Compound 19 – ^{13}C NMR ($CDCl_3$, 400 MHz)

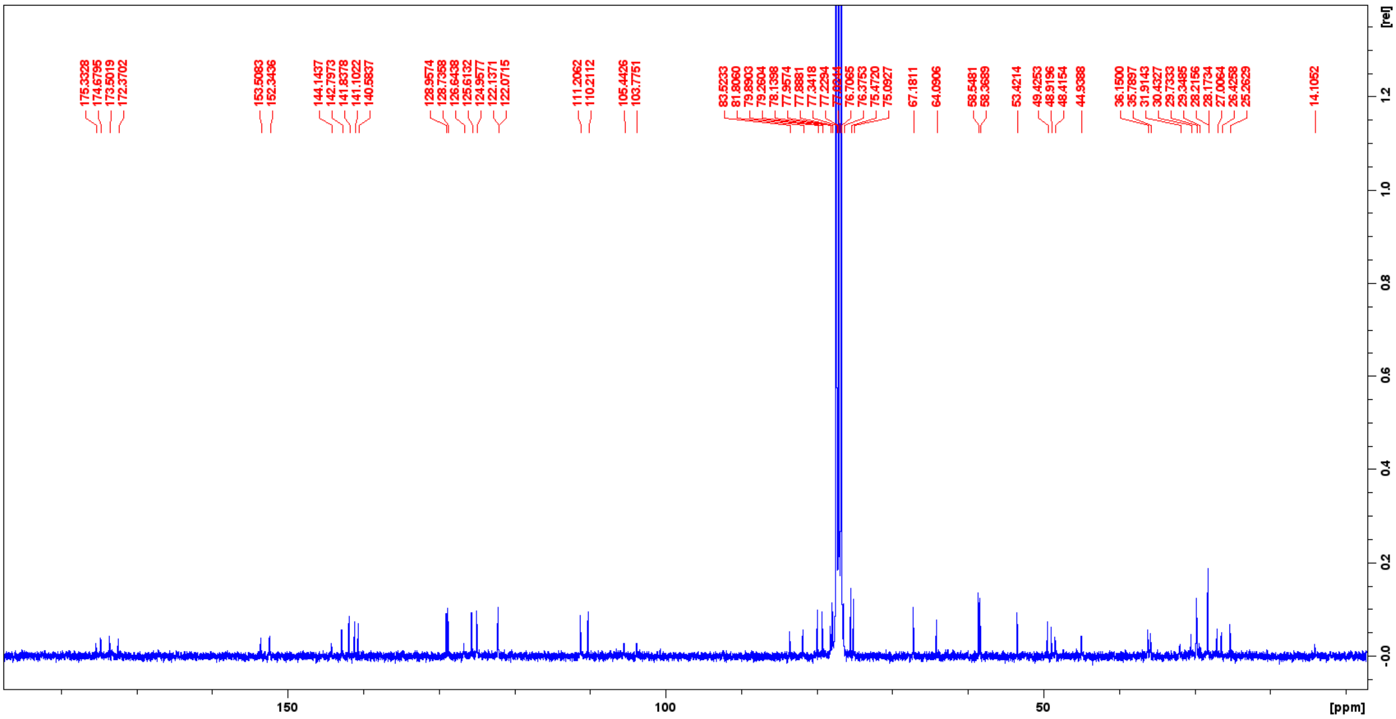

Compound 20 - ^{1}H NMR ($CDCl_3$, 400 MHz)

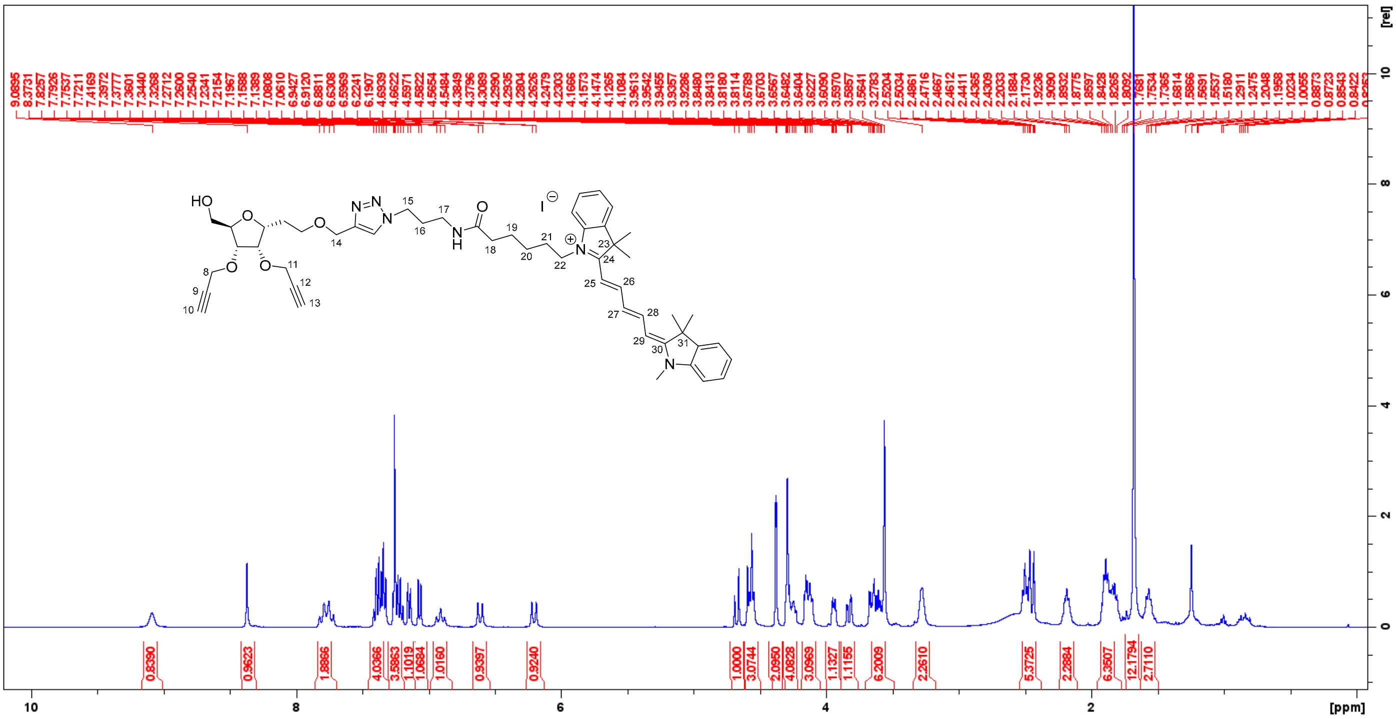

Compound 20 – ^{13}C NMR ($CDCl_3$, 100.6 MHz)

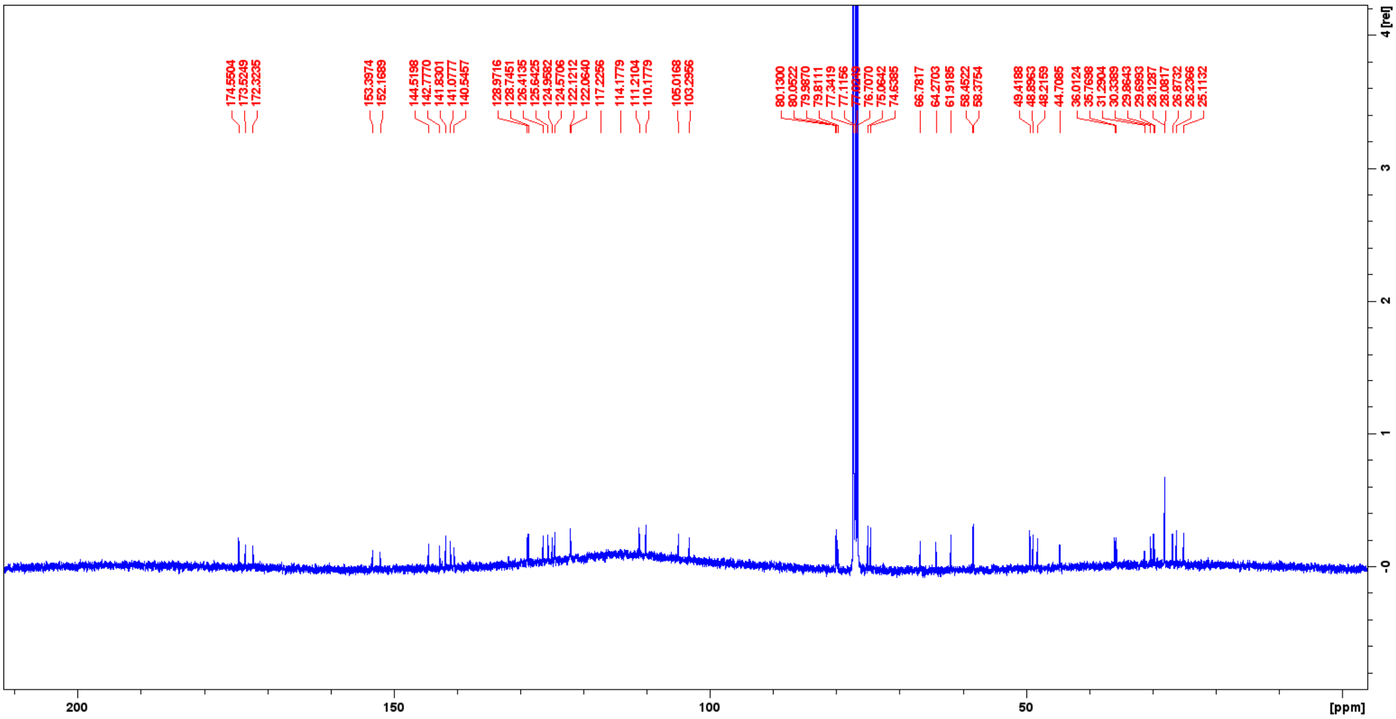

Compound 22 – ^{1}H NMR (D_2O, 400 MHz)

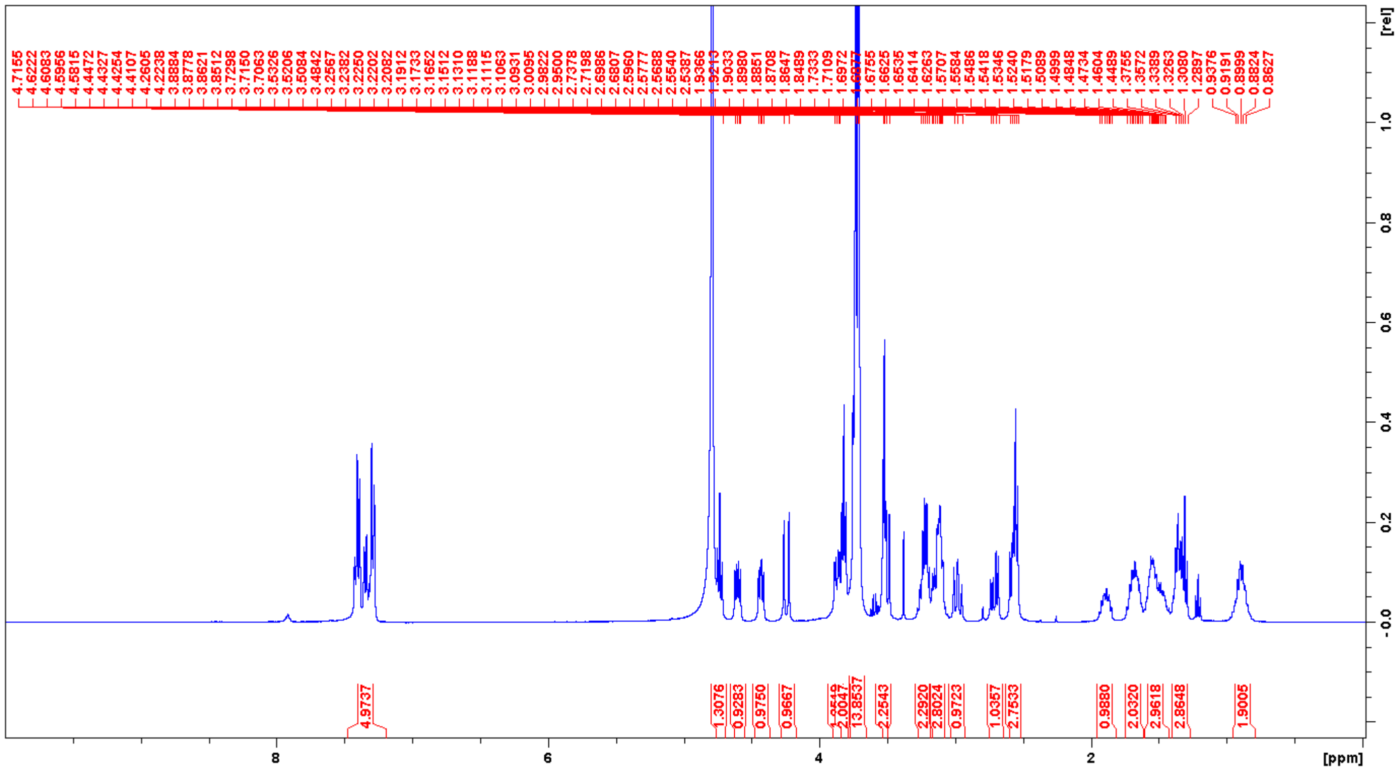

Compound 25 – ^1H NMR ($CDCl_3$, 400 MHz)

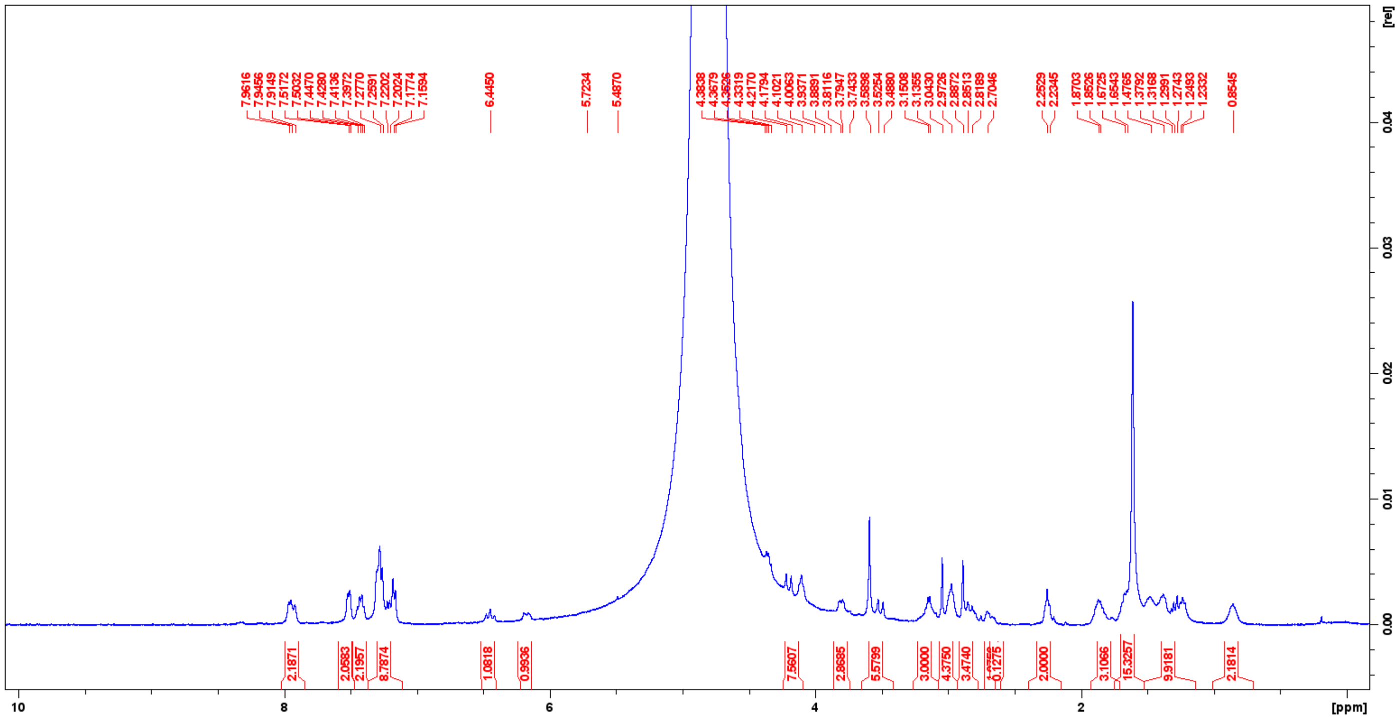

Compound 26 – ^{1}H NMR ($CDCl_3$, 400 MHz)

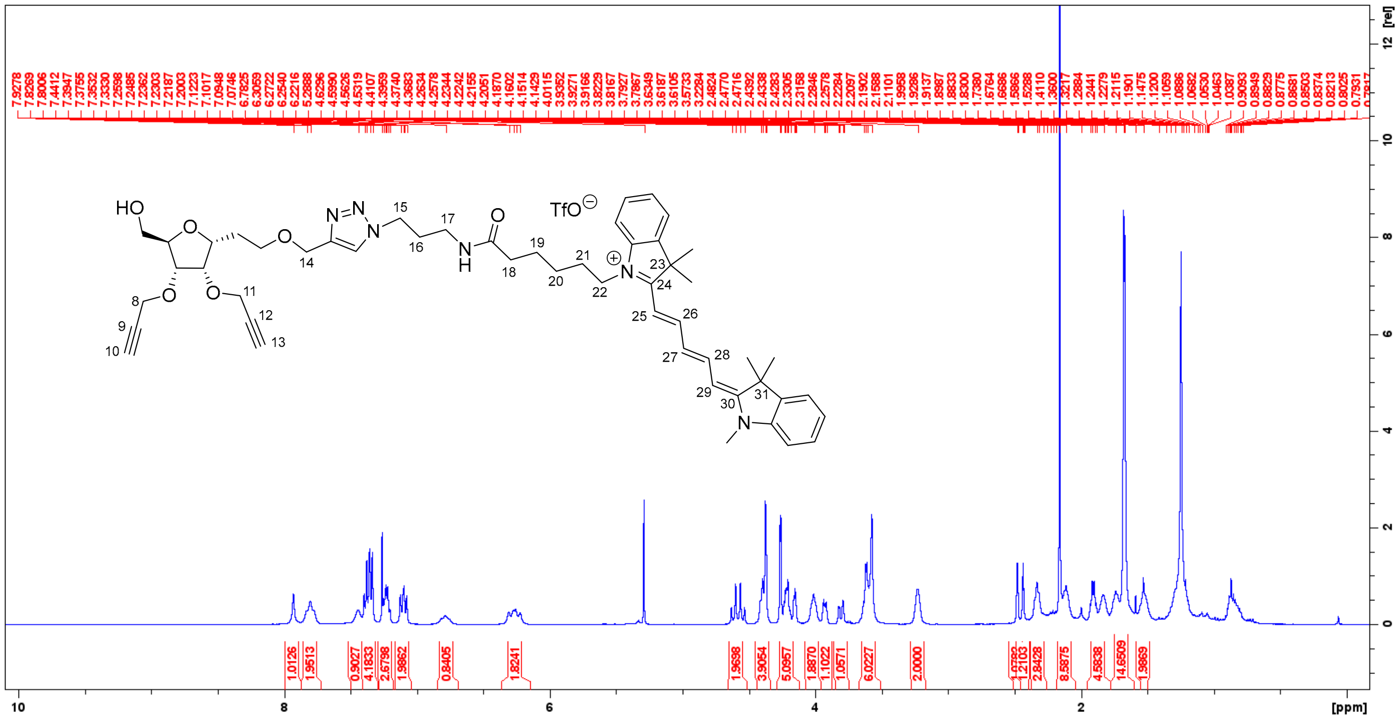

Compound 26 - ^{19}F NMR (CD_3CN, 376 MHz)

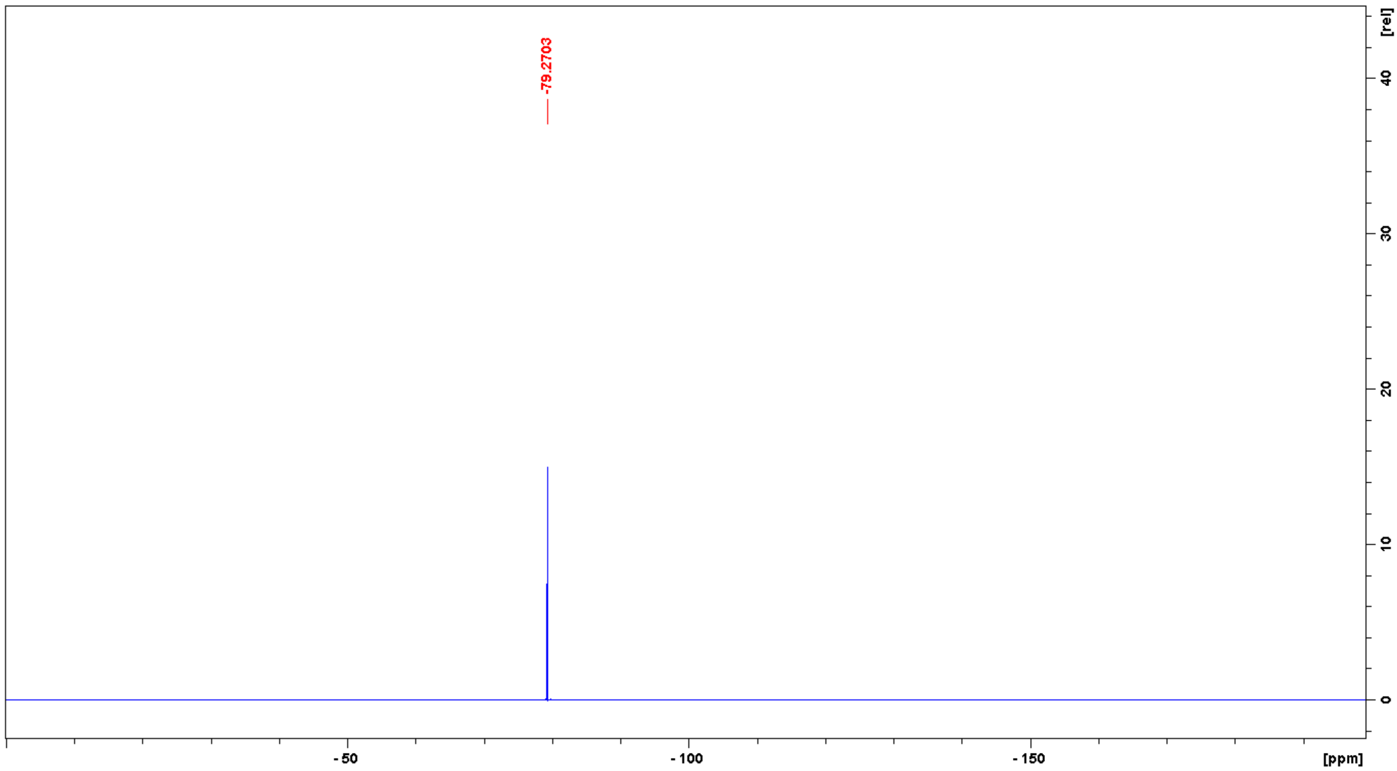

Compound 27 – ^{1}H NMR (CD_3CN, 400 MHz)

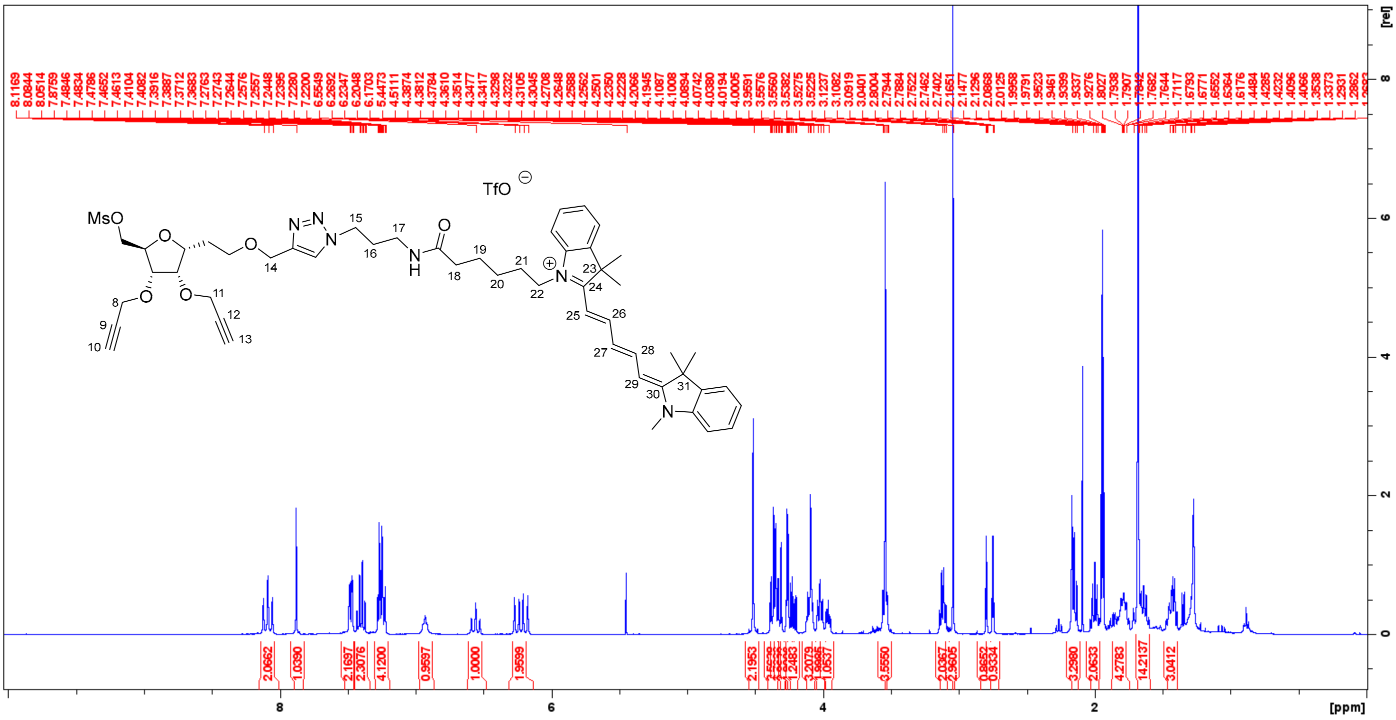

Compound 27 – ^{13}C NMR (CD_3CN, 100.6 MHz)

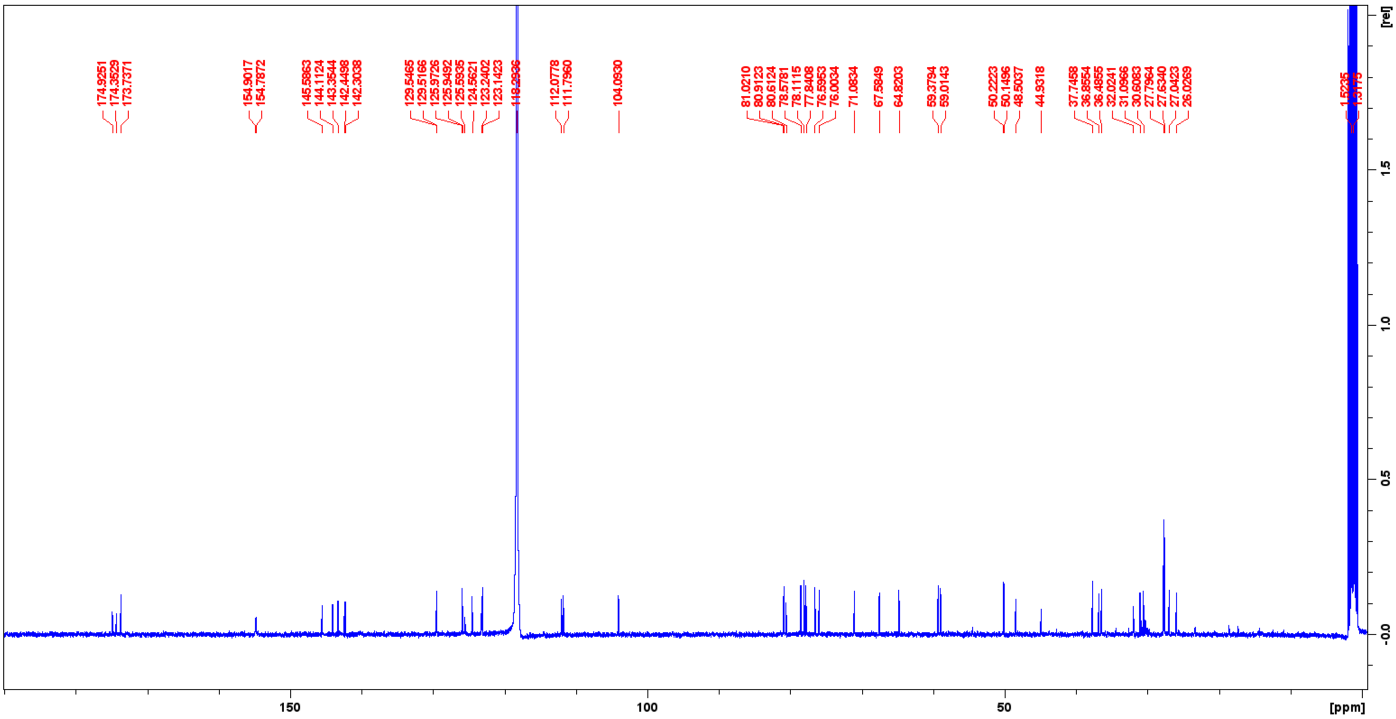

Compound 27- ^{19}F NMR (CD_3CN, 376 MHz)

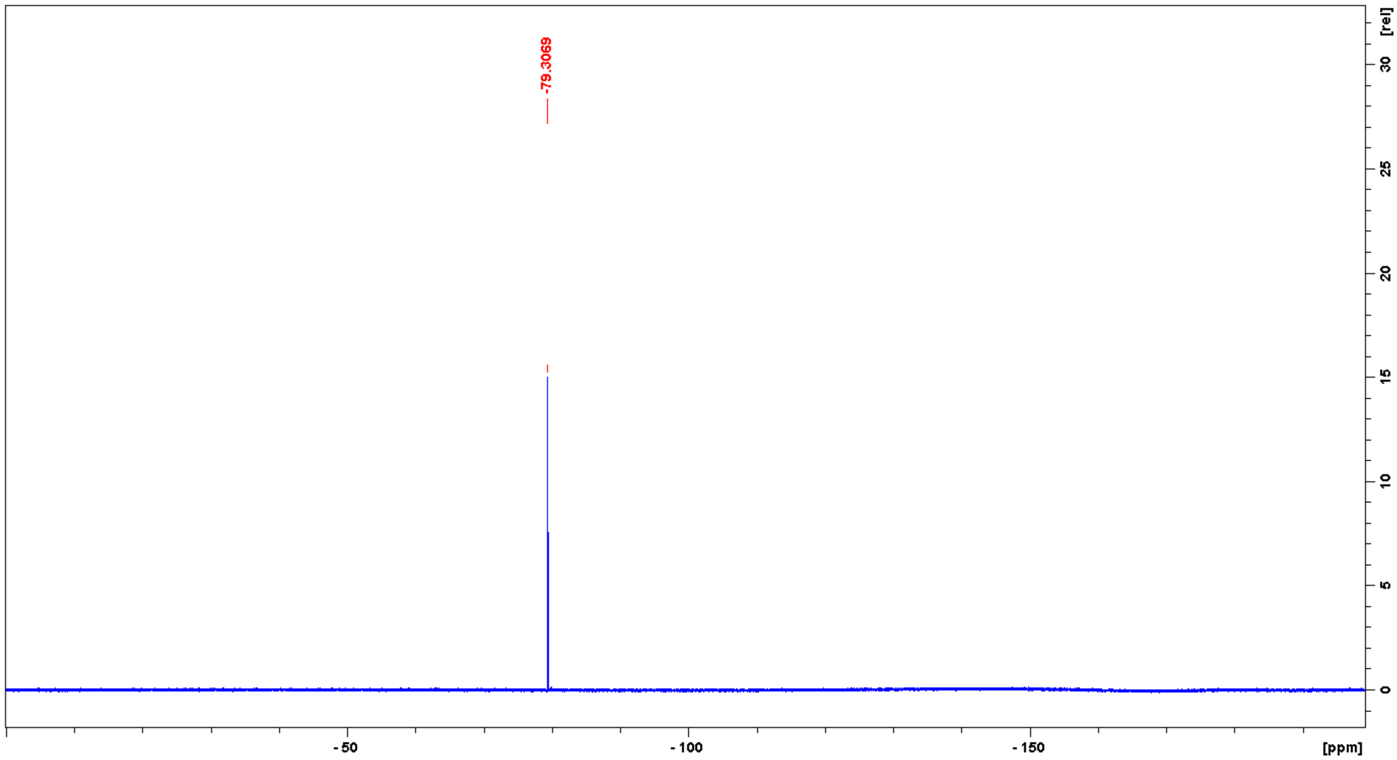

Copy of HRMS for compounds **21-22** and **23-24**

Compound **21**

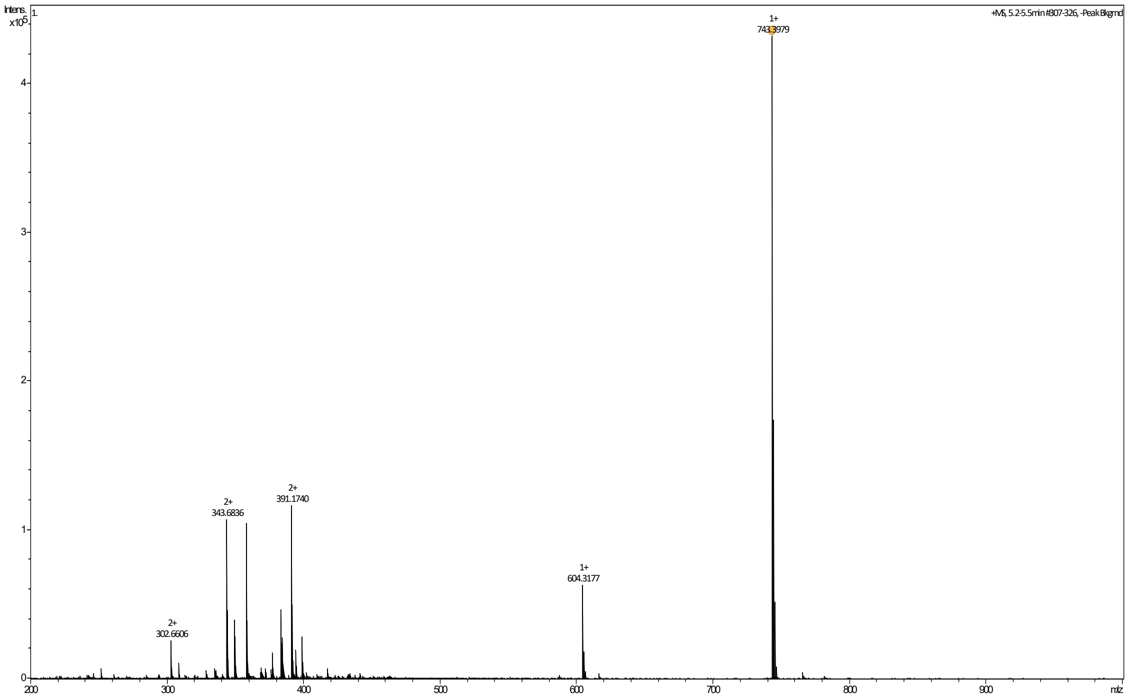

HRMS $[M]^+$ m/z = 743.3979 (calculated for $C_{33}H_{51}N_{12}O_8^+$: 743.3947). 3.2 mDa and 4.3 ppm

Compound **22**

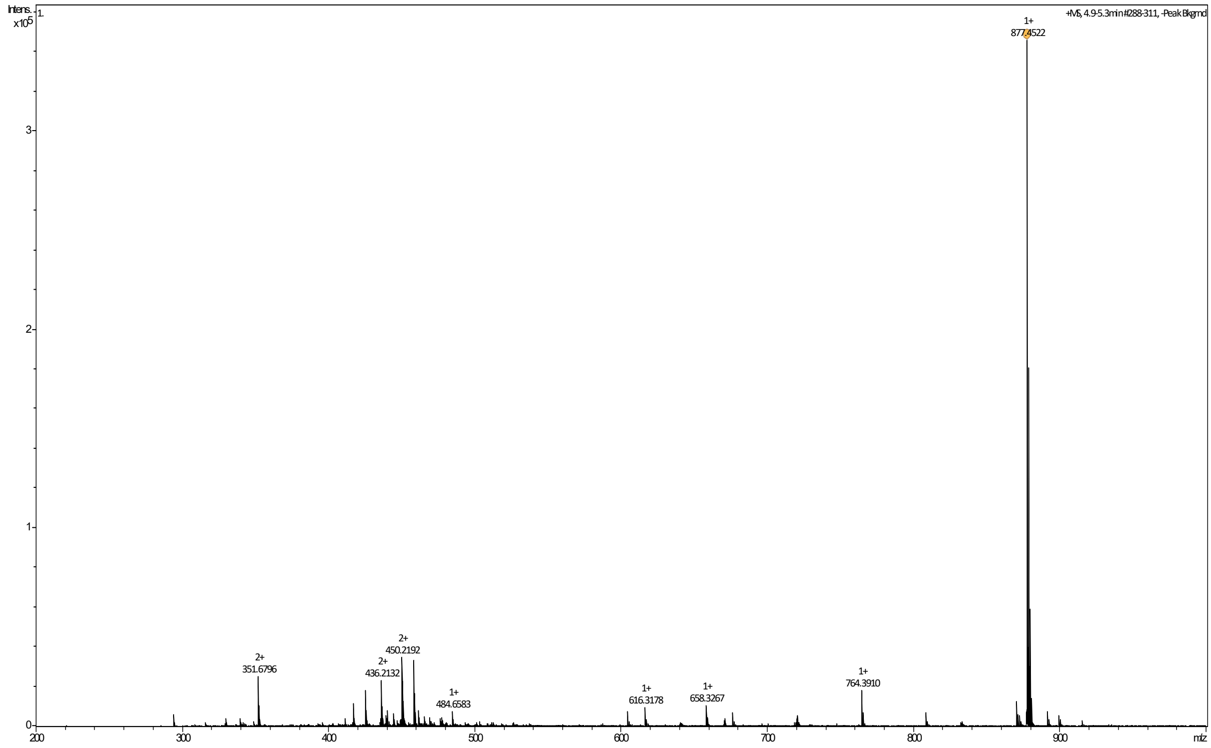

HRMS $[M]^+$ m/z = 877.4522 (calculated for $C_{38}H_{61}N_{12}O_{12}$: 877.4526).

Compound **23**

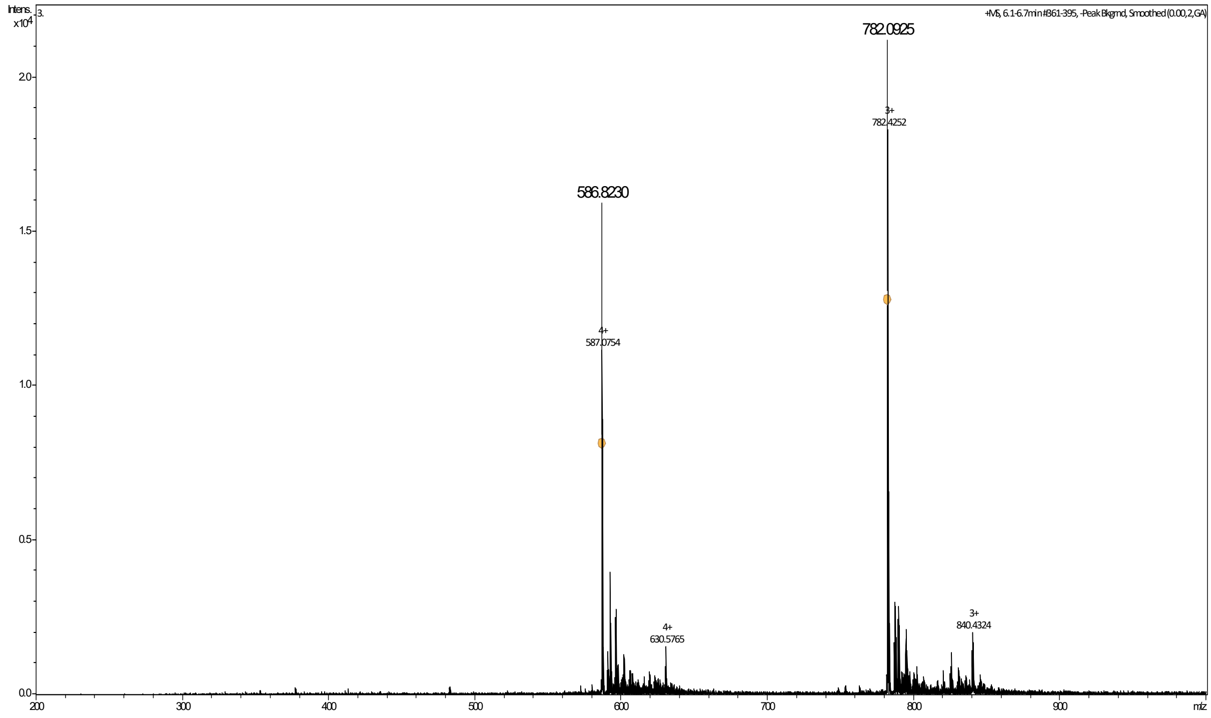

$[M+H]^{4+}$ m/z = 586.8230 (calculated for $C_{117}H_{167}FN_{30}O_{21}$: 586.8221). 1.2 mDa and 1.6 ppm

$[M]^{3+}$ m/z = 782.0925 (calculated for $C_{117}H_{166}FN_{30}O_{21}$: 782.0937). 0.69 mDa and 1.5 ppm

Compound **24**

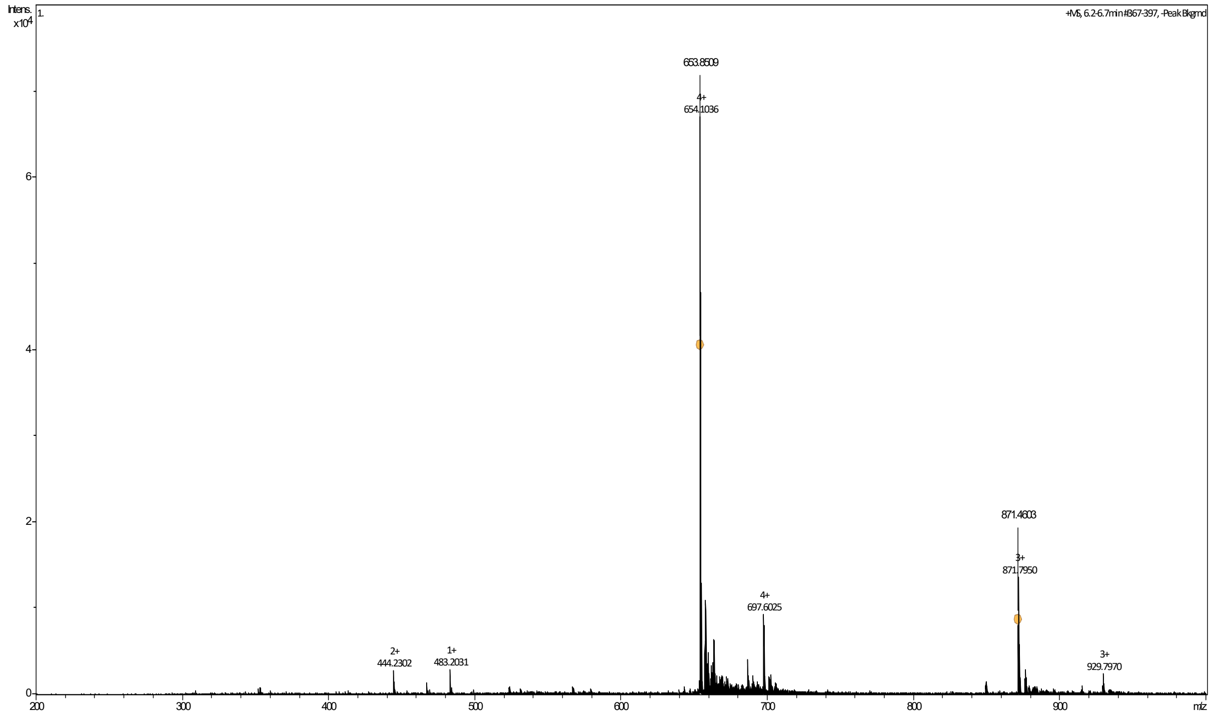

$[M+H]^{4+}$ m/z = 653.8509 (calculated for $C_{127}H_{187}FN_{30}O_{29}$: 653.8511). 5.3 mDa and 6.1 ppm

$[M]^{3+}$ m/z = 871.4603 (calculated for $C_{127}H_{186}FN_{30}O_{29}$: 871.4657). 0.2 mDa and 0.3 ppm

Analytical HPLC profile of compound **19** (Figures S1 and S2)

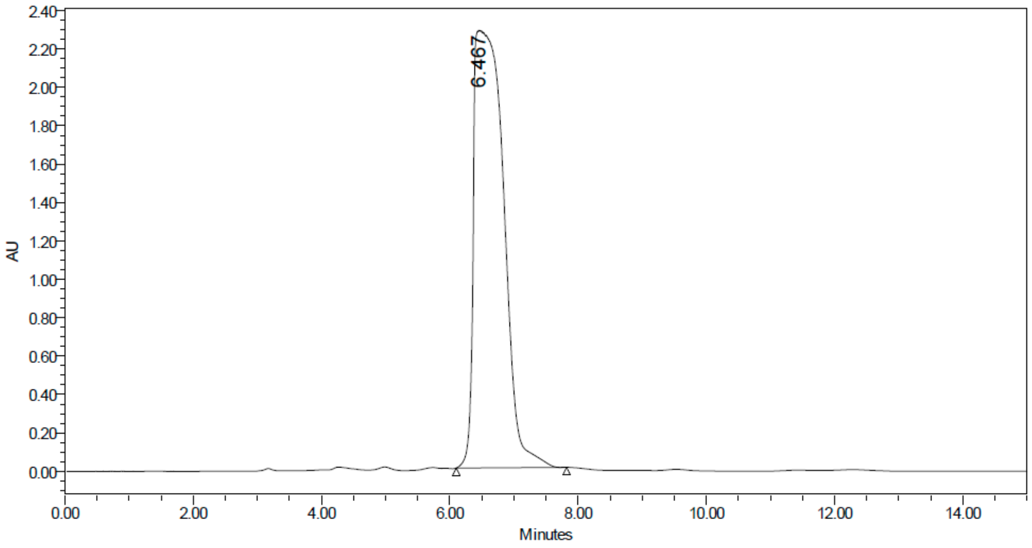

Figure S1. Analytical HPLC profile of **19** on Waters analytical HPLC. Retention time of: 6.5 min; Conditions: Luna PFP column eluted with ACN/H2O 60/40 (v/v) with 0.1% of TFA in isocratic conditions with a flow rate of 1.0 mL/min; UV detection (650 nm).

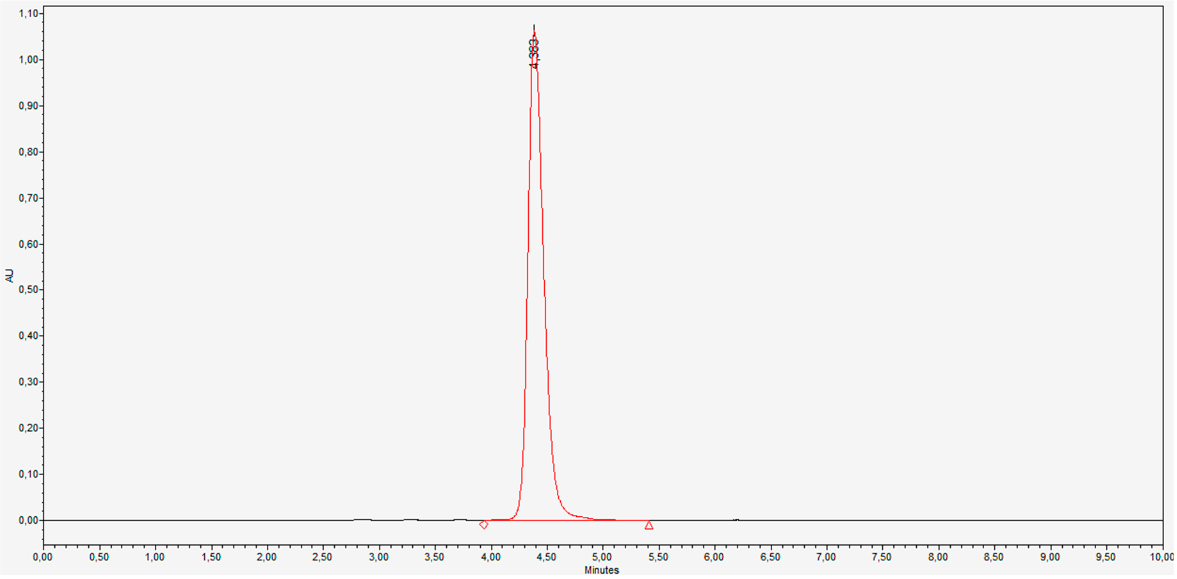

Figure S2. Analytical HPLC profile of **19** on Waters Alliance 2690. Retention time of: 4.4 min; Conditions: Luna PFP column eluted with ACN/H2O 60/40 (v/v) with 0.1% of TFA in isocratic conditions with a flow rate of 1.5 mL/min; UV detection (650 nm).

References

1) M. Richard, J. Ariztia, S. Lamandé-Langle, N. Pellegrini Moïse, *Chemistry Select*, **2018**, *3*(31), 9121-9126

2) Y. Li, Y. Liu, L. Zhang, Y. Xu, *J. Label Compd. Radiopharm,* **2012**, *55*, 229–234.
